# Prednisolone Once Daily vs Hydrocortisone Thrice Daily in Hypoadrenalism: A Randomized Clinical Trial

**DOI:** 10.1001/jamanetworkopen.2026.2982

**Published:** 2026-03-24

**Authors:** Sirazum Choudhury, Katharine Lazarus, Angelica Sharma, Kavita Narula, Cara Go, Suzie Cro, Thilipan Thaventhiran, Bernard Khoo, Tricia Tan, Karim Meeran

**Affiliations:** 1 Department of Endocrinology, Imperial College Healthcare NHS Trust, London, UK; 2 Division of Diabetes, Endocrinology and Metabolism, Department of Metabolism, Digestion, and Reproduction, Imperial College London, London, UK; 3 Department of Clinical Biochemistry, North West London Pathology, London, UK; 4 Imperial Clinical Trials Unit, Imperial College London, London, UK; 5 Endocrinology, Division of Medicine, University College London, London, UK

## Abstract

**Importance:**

Adrenal insufficiency is conventionally treated with daily multiple-dose hydrocortisone, and once-daily low-dose prednisolone is an alternative for glucocorticoid replacement. Clinical trials comparing once-daily low-dose prednisolone with thrice-daily hydrocortisone are lacking.

**Objective:**

To examine the differences in metabolism and bone turnover in patients receiving hydrocortisone vs prednisolone for adrenal insufficiency.

**Design, Setting, and Participants:**

This double-blind, crossover randomized clinical trial of multiple-daily standard-dose hydrocortisone vs once-daily low-dose prednisolone (2-5 mg), performed from September 3, 2019, to December 14, 2023, involved adults with adrenal insufficiency. Anthropometrics, biochemical data for cardiometabolic and bone health, and subjective health survey data were collected at days 1, 30, and 120 of each study period for both medications.

**Intervention:**

Individuals were randomized to receive 4 months of once-daily prednisolone in the morning (with placebos at noon and afternoon) or hydrocortisone at the same times. All participants were crossed over to the alternative treatment for an additional 4 months.

**Main Outcome and Measures:**

The primary outcome was assessment of bone turnover, detected by change in carboxylated and undercarboxylated osteocalcin between days 1 and 120 in each treatment period. Secondary outcomes included change in weight, body mass index, waist circumference, glycated hemoglobin, and subjective heath survey responses.

**Results:**

Forty-seven participants were randomized, with 46 participants included in the analysis (median [IQR] age, 55.0 [46.5-62.8] years; 24 [52.2%] male). Twenty-four received prednisolone first and 22 received hydrocortisone first. Bone turnover was significantly slowed with prednisolone compared with hydrocortisone as evidenced by a significantly lower level of multiple bone markers, including carboxylated osteocalcin (mean treatment difference, −1.22 ng/mL; 95% CI, −2.35 to −0.10 ng/mL; *P* = .04), undercarboxylated osteocalcin (mean treatment difference, −1.38 ng/mL; 95% CI, −2.32 to −0.44 ng/mL; *P* = .005) (to convert osteocalcin to micrograms per liter, multiply by 1), urinary N-terminal telopeptide (mean treatment difference, −9.34 nmol/mmol; 95% CI, −15.4 to −3.29 nmol/mmol; *P* = .002), and procollagen type 1 N-terminal propeptide (mean treatment difference, −13.8 ng/mL; 95% CI, −22.2 to −5.49 ng/mL; *P* < .001). The mean treatment group difference in weight reduction from baseline was −1.87 kg (95% CI, −3.02 to −0.72 kg; *P* = .002) for prednisolone treatment compared with hydrocortisone, and this was associated with concordant significantly greater reductions in body mass index (BMI; calculated as weight in kilograms divided by the square of height in meters; treatment difference, −0.522; 95% CI, −1.01 to −0.04; *P* = .04), waist circumference (treatment difference, −2.26 cm; 95% CI, −3.97 to −0.56 cm; *P* = .01), and HbA_1c _(treatment difference, −0.12% [−1.23 mmol/mol; 95% CI, −1.95 to −0.51 mmol/mol]; *P* = .001). There were no differences in safety measures or subjective health outcomes, including all 36-Item Short-Form Health Survey domains and Addison’s Disease-Specific Quality of Life Questionnaire.

**Conclusions and Relevance:**

In this randomized clinical trial, once-daily low-dose prednisolone was associated with slower bone turnover and improvements in cardiometabolic health markers compared with multiple-dose hydrocortisone without compromising well-being. Studies assessing longer-term mortality and morbidity outcomes are needed.

**Trial Registration:**

ClinicalTrials.gov Identifier: NCT03936517

## Key Points

**Question:**

Are there differences in bone turnover between once-daily low-dose prednisolone and multiple-daily standard-dose regimens of hydrocortisone on cardiometabolic outcomes in patients with adrenal insufficiency?

**Findings:**

In this randomized clinical trial of 46 adults with adrenal insufficiency, bone turnover was slowed with prednisolone treatment, which was also associated with reductions in body weight, waist circumference, and glycated hemoglobin. Bone formation and resorption markers were elevated with hydrocortisone treatment, despite no detectable differences in subjective health outcomes.

**Meaning:**

These results suggest that once-daily low-dose prednisolone is a safe alternative to standard regimens of hydrocortisone for treatment of adrenal insufficiency, which may have better cardiovascular outcomes.

## Teaser

This randomized clinical trial investigates how bone turnover in patients with adrenal insufficiency is affected by once-daily prednisolone compared with multiple-dose hydrocortisone treatment.

## Introduction

Glucocorticoid replacement is an essential lifesaving treatment for patients with adrenal insufficiency. The prevailing approach to management is to use daily multiple-dose regimens of hydrocortisone.^1^ In some countries, cortisone acetate is preferred. Alternatives include once-daily prednisolone (or prednisone in some countries) and once-daily dual-release hydrocortisone.^2^ Prednisone is extensively metabolized to prednisolone by hepatic 11β-hydroxysteroid dehydrogenase in first-pass hepatic metabolism.^3,4^ In the context of the Endocrine Society guidelines, hydrocortisone is recommended at a dose of 15 to 25 mg in 2 to 3 divided doses. Prednisolone is an alternative administered at 3 to 5 mg in 1 to 2 divided doses.^5^ More recently, the National Institute for Health and Care Excellence in the UK has reaffirmed these recommendations in 2024.^6^

Despite glucocorticoid replacement therapy, adrenal insufficiency is associated with an increased mortality.^7,8,9^ In addition to the risk of undertreatment and subsequent adrenal crises, this is possibly due to excess glucocorticoid exposure or replacement regimens that deviate from normal physiological cortisol profiles.^10,11^ There is no consensus on how to optimize replacement glucocorticoid therapy for primary or secondary adrenal insufficiency.^12^ Excess replacement, particularly later in the day, frequently occurs with conventional hydrocortisone therapy and is associated with increased bone loss, risk of cardiovascular deaths, and reduced quality of life.^13,14,15,16^

Prednisolone is rapidly absorbed, has a prolonged cellular effect, and has a longer half-life than hydrocortisone, enabling once-daily administration. Methylprednisolone is more potent than prednisolone and has not been addressed here. Low-dose prednisolone is superior in reducing androgens and 17-hydroxyprogesterone as well as improving growth velocity in patients with congenital adrenal hyperplasia compared with thrice-daily hydrocortisone.^17^ It is 6 to 8 times more potent than hydrocortisone. A replacement dose of 3 to 4 mg daily may therefore be equivalent to a total daily dose of 20 mg of hydrocortisone, which needs to be given in divided doses because of its short half-life. A pharmacokinetic study of prednisolone revealed that a mean dose of 3.86 mg once daily provides effective replacement in adrenal insufficiency, with a peak concentration at 1.43 hours.^18^ Prednisolone has the added advantage of being widely available. No comparative study has examined the effect of low-dose prednisolone (2-5 mg) once daily vs conventional multiple daily doses of hydrocortisone. We investigated how body weight, metabolic parameters, and bone turnover in patients with adrenal insufficiency are affected by once-daily prednisolone administration compared with multiple daily doses of hydrocortisone in a double-blind, crossover randomized clinical trial.

## Methods

### Study Design

This 9-month, 2-arm, 2-period, double-blind, crossover randomized clinical trial was undertaken at the National Institute for Health and Care Research Imperial Clinical Research Facility, Hammersmith Hospital, London, UK. The study ran from September 3, 2019, to December 14, 2023. The trial design and full methods can be found in the study protocol in Supplement 1. The trial was overseen by both an unblinded data monitoring committee and blinded trial steering committee and was conducted in accordance with the Medicines for Human Use (Clinical Trials) regulations 2004 (SI 2004/1031) and amended regulations (SI 2006/1928) and the International Conference on Harmonization Good Clinical Practice guidelines. Ethical review and approval were granted by the Medicines and Healthcare Products Regulatory Agency and the UK Health Research Authority National Research Ethics Service. All participants provided written informed consent. The Consolidated Standards of Reporting Trials (CONSORT) reporting guideline was followed.

Briefly, participants were randomized 1:1 to receive prednisolone or hydrocortisone replacement therapy in the first 120-day study period, followed by the alternative treatment in the second 120-day study period. Baseline data were collected on the first day of each study period and end point data on days 30 and 120. The 2 study periods were separated by a minimum 2-week washout period during which participants returned to open-label replacement therapy. The assay platforms used in this study and the performance specifications for each analyte can be found in eTable 1 in Supplement 2.

### Participants

Adults aged 18 to 70 years with primary or secondary adrenal insufficiency for at least 6 months were recruited if they were undergoing stable hormone replacement therapy, including glucocorticoid replacement (hydrocortisone or prednisolone), for the preceding 3 months with no alteration of drug or dose. None of the participants had glucocorticoid-induced adrenal insufficiency or were treatment naive. The causes of adrenal insufficiency in the cohort are specified in eTable 2 in Supplement 2. Individuals with a diagnosis of diabetes mellitis or congenital adrenal hyperplasia or who were taking medication that might interfere with glucocorticoid steroid metabolism were excluded. Verbal advice was given to all participants to take their tablets on an empty stomach (avoiding food for 1 hour before and after dosing if possible). This is our usual clinical practice based on evidence that food interferes with prednisolone absorption.^19^

### Randomization and Masking

Participants were randomized to 1 of 2 trial arms after blocking for the type of adrenal insufficiency (primary vs secondary). Randomization was managed using the Oracle InForm electronic data capture application. An original randomization list was populated by the study statistician (S. Cro) and was uploaded to the electronic capture application, which generated study drug identification numbers, which were used to blind the trial management group and could be decoded by the trial pharmacy to enable them to dispense the correct medication. The study statistician (S. Cro) did not have any further involvement in the running of the trial but was a member of the unblinded data monitoring committee. Participants were enrolled onto the study by S. Choudhury, who randomized each participant using the electronic capture application, which generated the drug identification numbers.

Throughout the trial, participants, treating physicians, and outcome assessors were blinded to treatment arm. To maintain blinding, placebo tablets were administered at noon and 4 pm in patients who were taking a single morning tablet containing 2.0 to 5.0 mg of prednisolone during the prednisolone period. In the hydrocortisone period, patients received a single tablet of 10.0 to 20.0 mg of hydrocortisone in the morning followed by 2.5 to 10.0 mg at noon and 2.5 to 5.0 mg in the afternoon. The daily tablet count was therefore the same regardless of whether the patient was receiving prednisolone or hydrocortisone. For the sick day rules, all patients were asked to double their doses by initially having an extra dose of the morning tablet in addition to the scheduled tablet due. Subsequent doses in the doubling regimen involved taking 2 tablets instead of 1 from the scheduled bottle. This ensured that all participants had at least doubled the first dose if they were taking hydrocortisone and had doubled for the day if taking prednisolone. All tablets were manufactured specifically for the trial and bottled by Activase Pharmaceuticals Ltd and labeled by Tiofarma B.V.

### Procedures

The dose of prednisolone or hydrocortisone used in the main study was determined by the pretrial regimen already used by each participant. For instance, if a participant had been receiving 10 mg plus 5 mg plus 5 mg of hydrocortisone stably for 3 months before recruitment, then this regimen was taken forward into the blinded portion of the trial. Participants naive to 1 of the 2 glucocorticoids were given a trial of the drug they had not experienced before randomization. Where a participant was naive to hydrocortisone, they underwent a trial of 10.0 mg plus 5.0 mg plus 2.5 mg daily. Where a participant was naive to prednisolone, they underwent a trial of 3 or 4 mg once daily.^4^ Their final study regimen for the trialed drug was determined by titration according to standard clinical care used at Imperial College Healthcare NHS Trust (eMethods in Supplement 2). Before randomization, patients confirmed they were comfortable with both regimens and progressed into the blinded study.

Participants were invited to a baseline visit on day 1, where the study medication was dispensed. Outcome data were collected on days 30 and 120. This was repeated for both medications before and after crossing over. Each study visit was completed in the morning at the same time for each participant on each occasion to minimize diurnal effects. Participants were instructed to fast overnight and to take their morning glucocorticoid tablet at a fixed time, allowing for blood samples to be collected at 2 hours after dosing. The initial screening visit was conducted in a similar manner to allow for the participant to acclimatize to the process before the study data collection began in earnest. Each visit was regimented, with the participant providing a urine sample on arrival, after which they were weighed and waist and hip circumferences measured. Waist and hip circumferences were measured in accordance with suggested approach published by the World Health Organization.^20^ The hips were defined as the widest part of the buttocks from a rear profile. The waist was the midpoint between the lowest palpable ribs and the iliac crest. The subjective health surveys were then completed, with blood pressure and heart rate measured immediately afterward with the patient sitting, representing resting values. Blood samples were then collected at 2 hours after dosing before the patient was discharged. Participants received telephone calls from the trial management group at fixed intervals for safety and adverse event reporting.

### Outcomes

The primary outcome was assessment of bone turnover, detected by change in carboxylated osteocalcin and under-carboxylated osteocalcin between days 1 and 120 in each treatment period. Secondary outcomes measured throughout 120 days included longer-term markers of bone turnover, blood pressure, heart rate, weight, body composition, and waist-hip circumferences. Weight and body composition were measured using bioimpedance (Tanita Corporation of American Inc). Glycemic control was assessed using change in glycated hemoglobin (HbA_1c_). Fractional excretion in urine was calculated from spot urine samples. Subjective health data were collected using the 36-Item Short-Form Health Survey (SF-36) and Addison’s Disease-Specific Quality of Life Questionnaire (Addi-QoL). The Addi-QoL is validated to assess quality of life in patients with adrenal insufficiency and scores 30 items on a range of 1 to 4, with higher scores indicating better outcomes.^21^ The other secondary outcomes are outlined in the eMethods in Supplement 2.

### Sample Size

To detect a difference of 1.5 ng/mL in osteocalcin (to convert to micrograms per liter, multiply by 1) with a 2-sided significance (type I error) rate of 5%, assuming an estimate of the SD at a single time point of 3.0 with 90% power, 44 completers were required. This assumed cautiously that the correlation between the repeated measurements within patients across the period is 0.5, whereas a previous crossover trial^5^ showed a correlation of 0.8 (Toby Prevost, PhD, Imperial College, personal oral communication, January 13, 2017).

### Statistical Analysis

Outcome measures and baseline data were assessed for normality using the Shapiro-Wilk test. Approximately normally distributed data have been reported as mean (SD) and nonnormal data as median (IQR). Analysis was conducted based on the intention-to-treat principle. Outcome measures were analyzed using a multilevel, repeated-measures, linear, mixed-effect model. For each outcome this included the participant as a random effect and the treatment, period, type of adrenal insufficiency (primary vs secondary), baseline value of the outcome, and concurrent use of relevant medication (eg, bone-sparing agents in the case of bone markers) as fixed effects. The model for each outcome was fitted separately to data at days 30 and 120 to assess the treatment effects at both time points (with 95% CIs).

A 2-sided *P* < .05 was regarded as significant for the primary outcome. *P* values are also presented for secondary outcomes but should be interpreted cautiously because adjustment for multiplicity has not been applied in line with the trial’s prespecified statistical analysis plan (Supplement 1). Analysis was conducted using R software, version 4.4.2 (R Foundation for Statistical Computing).

## Results

**Figure 1.**
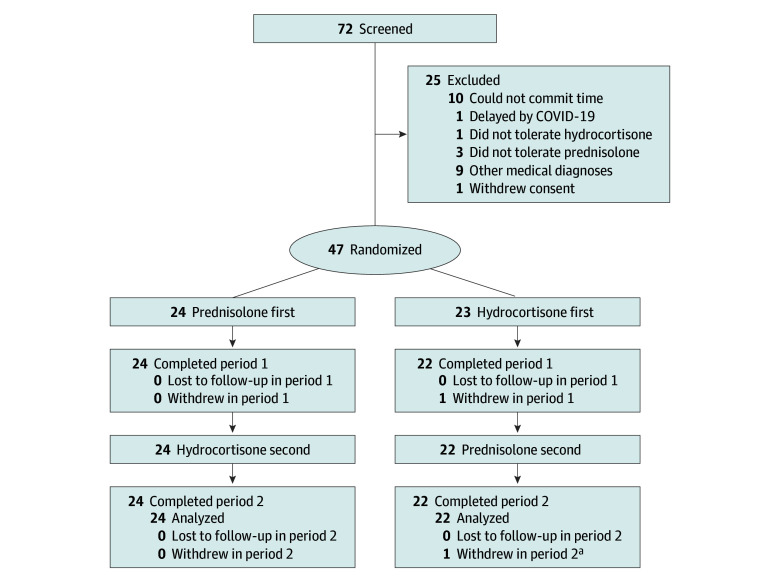
CONSORT Diagram of Trial Profile CONSORT indicates Consolidated Standards of Reporting Trials. ^a^Participant was converted to open-label hydrocortisone after a serious adverse event after the day 30 visit in period 2. He completed all study events thereafter and was included in the final analysis as per the intention-to-treat analysis.

Forty-seven participants were randomized to this study between September 3, 2019, and December 13, 2022, with the last patient visit on December 14, 2023. Forty-six participants were included in the analysis (median [IQR] age, 55.0 [46.5-62.8] years; 24 [52.2%] male and 22 [47.8%] female), including 16 with primary adrenal insufficiency (Figure 1). One participant decided to withdraw after the baseline visit on day 1 and did not receive any study medication. No outcome data were collected on this individual, and they were therefore excluded from all analyses. There were no differences in the prerandomization baseline demographics between the 2 arms (Table 1). There were also no significant differences at baseline between the treatment arms for the rate and severity of infections, routine hematology and biochemistry panels, or well-being as assessed by subjective health questionnaires. All participants had been receiving glucocorticoid replacement therapy for at least 2 years. All participants with primary adrenal insufficiency were receiving fludrocortisone at their usual doses, which were unchanged from their prestudy regimen. Doses were not modified in the study and are reported in Table 1. The median (IQR) dose of hydrocortisone used in the study was 20.0 (17.5-20.0) mg vs 3.5 (3.0-4.0) mg for prednisolone. A breakdown of the frequency of regimens can be found in eTable 3 in Supplement 2.

**Table 1.** Baseline Characteristics of the Study Patients^a^

Characteristic	No. (%) of participants^b^		
	All participants (N = 46)	Prednisolone-first arm (n = 24)	Hydrocortisone-first arm (n = 22)
Age, median (IQR), y	55.0 (46.5-62.8)	51.5 (42.8-63.2)	57.5 (48.2-62.0)
Sex			
Female	22 (47.8)	10 (41.7)	12 (54.5)
Male	24 (52.2)	14 (58.3)	10 (45.5)
Adrenal insufficiency			
Primary	16 (34.8)^c^	8 (33.3)	8 (36.4)
Secondary	30 (65.2)^d^	16 (66.6)	14 (63.6)
Baseline steroid			
Hydrocortisone	30 (65.2)	13 (54.2)	17 (77.3)
Prednisolone	16 (34.8)	11 (45.8)	5 (22.7)
Baseline steroid dose, median (IQR)			
Hydrocortisone equivalent, mg	20 (20-21)	20 (20-21.4)	20 (17.5-20.75)
Prednisolone, mg	3 (3-4)	3 (3-4)	3 (3-4)
Hydrocortisone, mg	20 (20-20)	20 (20-20)	20 (17.5-20)
Fludrocortisone primary adrenal insufficiency, μg	100 (100-150)^e^	100 (100-112.5)^f^	118.75 (87.5-150)^f^
Therapy			
Bone antiresorptive therapy	6 (13.0)	2 (8.3)	4 (18.2)
Antihypertensives	7 (15.2)	3 (12.5)	4 (18.2)
Lipid-lowering agents	12 (26.1)	4 (16.7)	8 (36.4)
Weight, mean (SD), kg	79.0 (18.5)	81.6 (20.6)	76.3 (15.9)
BMI, median (IQR)	25.9 (23.1–30.0)	26.5 (22.7-31.8)	24.9 (23.3-29.6)
Waist circumference, mean (SD), cm	93.1 (14.2)	93.9 (15.7)	92.2 (12.6)
Waist-hip ratio, mean (SD)	0.89 (0.08)	0.89 (0.08)	0.88 (0.08)
Pulse, mean (SD), beats/min	63.3 (11.1)	65.4 (12.8)	61.1 (8.7)
Systolic blood pressure, mean (SD), mm Hg	121.3 (15.1)	120.5 (16.3)	122.2 (14.1)
Diastolic blood pressure, mean (SD), mm Hg	73.9 (9.5)	73.0 (10.0)	74.9 (9.0)
Carboxylated osteocalcin, median (IQR), ng/mL	15.2 (13.4-17.3)	14.7 (13.6-17.2)	15.8 (13.3-20.4)
Undercarboxylated osteocalcin, median (IQR), ng/mL	4.9 (3.5-7.5)	5.0 (3.9-7.1)	4.7 (3.1-7.8)
Urinary N-terminal telopeptide, median (IQR), nmol/mmol	39.0 (26.0-51.8)	27.5 (25.8-49.8)	43.5 (30.5-59.3)
Procollagen type 1 N-terminal propeptide, median (IQR), ng/mL	40.9 (31.9-54.8)^g^ [n = 45]	37.5 (29.7-60.0)^h^ [n = 24]	42.1 (34.3-68.6)^i^ [n = 21]
Vitamin D, mean (SD), ng/mL	34.13 (12.62)	33.85 (12.90)	34.2 (12.54)
Glycated hemoglobin, mean (SD), mmol/mol	36.4 (3.4)	36.3 (3.3)	36.6 (3.5)
Glucose, mean (SD), mg/dL	88.29 (7.21)	88.29 (5.41)	88.29 (9.01)
Insulin, median (IQR), μIU/mL	5.3 (3.8-8.8)	5.4 (3.8-7.5)	5.3 (4.3-8.8)
HOMA-IR score, mean (IQR)	1.2 (0.8-1.9)	1.1 (0.8-1.6)	1.2 (0.9-1.9)
Addi-QoL score, median (IQR)	98.0 (88.3-104.5)	98.0 (88.8-103.5)	98,0 (88.8-104.5)

Abbreviations: Addi-QoL, Addison’s Disease-Specific Quality of Life Questionnaire; BMI, body mass index (calculated as weight in kilograms divided by the square of height in meters); HOMA-IR, Homeostatic Model Assessment for Insulin Resistance.

SI conversion factors: To convert glucose to millimoles per liter, multiply by 0.0555; insulin to picomoles per liter, multiply by 6.945; osteocalcin to micrograms per liter, multiply by 1; and vitamin D to nanomoles per liter, multiply by 2.496.

^a^ All summary measures are comparable between the prednisolone first and hydrocortisone first arms. Prednisolone dose and hydrocortisone dose refer to the baseline doses of these glucocorticoids used by participants before randomization. Prednisolone doses were converted to hydrocortisone using a ratio of 1:7.^17^

^b^ Unless otherwise indicated.

^c^ 10 Addison disease and 6 adrenalectomy.

^d^ 25 after pituitary surgery and 5 hypopituitarism.

^e^ 16 participants.

^f^ 8 participants.

^g^ 45 participants.

^h^ 24 participants.

^i^ 21 participants.

Primary outcome data and pertinent secondary outcome data are presented in Table 2. Other exploratory outcomes are reported in the eTable 4 in Supplement 2. No evidence of carryover effect was detected.

**Table 2.** Study Outcomes^a^

Outcome	Day 30				Day 120			
	Hydrocortisone treatment effect from baseline, estimated marginal mean (95% CI)	Prednisolone treatment effect from baseline, estimated marginal mean (95% CI)	Difference of treatment effect (prednisone – hydrocortisone) , estimated marginal mean (95% CI)	*P* value	Hydrocortisone treatment effect from baseline, estimated marginal mean (95% CI)	Prednisolone treatment effect from baseline, estimated marginal mean (95% CI)	Difference of treatment effect (prednisone – hydrocortisone), estimated marginal mean (95% CI)	*P* value
Carboxylated osteocalcin, ng/mL	0.40 (−0.51 to 1.31)	−0.44 (−1.35 to 0.47)	−0.84 (−1.84 to 0.17)	.10	0.36 (−0.67 to 1.39)	−0.87 (−1.90 to 0.17)	−1.22 (−2.35 to −0.10)	.04
Undercarboxylated osteocalcin, ng/mL	0.13 (−0.45 to 0.71)	−0.66 (−1.24 to −0.08)	−0.79 (−1.42 to 0.16)	.02	0.62 (−0.24 to 1.47)	−0.76 (−1.62 to 0.10)	−1.38 (−2.32 to −0.44)	.005
Urinary N-terminal telopeptide, nmol/mmol	1.40 (−3.64 to 6.45)	−2.34 (−7.37 to 2.68)	−3.78 (−9.37 to 1.8)	.18	6.42 (0.85 to 12.00)	−2.92 (−8.46 to 2.62)	−9.34 (−15.4 to −3.29)	.002
Procollagen type 1 N-terminal propeptide, ng/mL	2.64 (−1.48 to 6.76)	−3.44 (−7.56 to 0.68)	−6.08 (−10.6 to −1.59)	.009	7.62 (0.00 to 15.25)	−6.21 (−13.84 to 1.41)	−13.8 (−22.2 to −5.49)	<.001
Vitamin D, ng/mL	−2.79 (−6.30 to 0.72)	2.38 (−1.14 to 5.91)	5.17 (0.32 to 10.0)	.04	−4.12 (−9.45 to 1.21)	5.54 (0.19 to 10.90)	9.66 (2.28 to 17.0)	.01
Bone-specific alkaline phosphatase, U/L	−0.72 (−1.82 to 0.38)	0.19 (−0.91 to 1.29)	0.91 (−0.24 to 2.07)	.12	0.05 (−1.58 to 1.68)	0.037 (−1.59 to 1.66)	−0.13 (−1.75 to 1.73)	.99
Parathyroid hormone, pg/mL	−0.25 (−0.84 to 0.33)	−0.26 (−0.84 to 0.32)	−0.01 (−0.53 to 0.51)	.97	−0.18 (−0.83 to 0.48)	0.36 (−0.30 to 1.01)	0.53 (−0.12 to 1.2)	.11
Corrected calcium, mg/dL	−0.08 (−0.04 to 0.20)	0.12 (−0.04 to 0.02)	0.00 (−0.08 to 0.12)	.77	0.72 (−0.04 to 0.02)	0.04 (−0.08 to 0.16)	−0.04 (−0.15 to 0.08)	.45
Glycated hemoglobin, mmol/mol	−0.98 (−2.71 to 0.74)	−1.37 (−3.09 to 0.36)	−0.385 (−2.77 to 2.0)	.75	0.57 (0.05 to 1.08)	−0.66 (−1.18 to −0.15)	−1.23 (−1.95 to −0.51)	.001
Glucose, mg/dL	−1.62 (−3.60 to 0.36)	−1.44 (−3.42 to 0.54)	0.18 (−2.34 to 2.70)	.90	−1.98 (−3.78 to 0.00)	−0.54 (−2.52 to 1.44)	1.44 (−1.08 to 3.96)	.28
Insulin, μIU/mL	0.20 (−0.76 to 1.16)	−0.12 (−1.08 to 0.84)	−0.32 (−1.38 to 0.75)	.55	−0.03 (−0.93 to 0.88)	0.14 (−0.76 to 1.04)	0.17 (−1.0 to 1.34)	.77
HOMA-IR score	−1.14 (−2.17 to −0.11)	−0.40 (−1.43 to 0.63)	0.74 (−0.68 to 2.15)	.30	−1.00 (−3.01 to 1.01)	0.85 (−1.16 to 2.86)	1.85 (−0.92 to 4.62)	.19
C-peptide, ng/mL	84.89 (−59.21 to 229.0)	−2.90 (−147.13 to 141.09)	−87.92 (−237.76 to 62.24)	.25	79.15 (−83.99 to 242.60)	29.00 (−134.44 to 192.15)	−50.45 (−263.14 to 162.24)	.64
Fructosamine, mg/L	−0.11 (−1.36 to 1.15)	0.68 (−0.58 to 1.94)	0.78 (−0.73 to 2.29)	.30	0.32 (−0.90 to 1.55)	−0.14 (−1.36 to 1.09)	−0.46 (−1.65 to 0.73)	.44
HOMA β score	−98.5 (−208.0 to 11.4)	−11.0 (−121.0 to 98.8)	87.4 (−64.2 to 239)	.25	−97.3 (−236.0 to 41.6)	25.6 (−113.0 to 164.4)	123.0 (−68.7 to 314)	.20
Weight, kg	0.38 (0.01 to 0.75)	−0.51 (−0.87 to −0.13)	−0.88 (−1.4 to −0.37)	.001	0.83 (−0.01 to 1.66)	−1.04 (−1.88 to −0.21)	−1.87 (−3.02 to −0.72)	.002
Fat mass, kg	−0.18 (−0.51 to 0.15)	−0.22 (−0.55 to 0.11)	−0.04 (−0.49 to 0.42)	.87	0.08 (−0.55 to 0.72)	−0.74 (−1.38 to −0.11)	−0.83 (−1.70 to 0.05)	.06
Lean mass, kg	0.46 (0.07 to 0.84)	−0.27 (−0.65 to 0.11)	−0.73 (−1.25 to −0.21)	.007	0.65 (0.23 to −1.07)	−0.27 (−0.70 to 0.15)	−0.92 (−1.50 to −0.34)	.003
Water mass, kg	0.45 (0.11 to 0.78)	−0.24 (−0.58 to 0.09)	−0.69 (−1.12 to −0.25)	.002	0.68 (0.30 to 1.06)	−0.26 (−0.62 to 0.12)	−0.94 (−1.47 to −0.41)	<.001
BMI	−0.08 (−0.32 to 0.15)	−0.22 (−0.46 to 0.1)	−0.139 (−0.46 to 0.18)	.39	0.12 (−0.23 to 0.47)	−0.41 (−0.76 to −0.06)	−0.522 (−1.01 to −0.04)	.04
Waist circumference, cm	0.08 (−0.83 to 0.99)	−0.19 (−1.09 to 0.72)	0.27 (−1.52 to 0.98)	.67	1.14 (−0.09 to 2.38)	−1.12 (−2.36 to 0.11)	−2.26 (−3.97 to −0.56)	.01
Waist-hip ratio	0.00 (−0.01 to 0.01)	0.00 (−0.01 to 0.01)	0.00 (−0.02 to 0.01)	.51	0.00 (−0.01 to 0.01)	−0.01 (−0.02 to 0.00)	−0.01 (−0.02 to 0.00)	.12
Pulse, beats/min	−1.05 (−3.11 to 1.01)	−1.97 (−4.03 to 0.09)	−0.92 (−3.71 to 1.87)	.51	2.15 (−0.36 to 4.66)	−0.95 (−3.45 to 1.56)	−3.1 (−6.52 to 0.33)	.07
Systolic blood pressure, mm Hg	1.15 (−1.87 to 4.14)	0.56 (−2.43 to 3.55)	−0.59 (−4.75 to 3.56)	.78	3.18 (0.24 to 6.12)	0.244 (−2.66 to 3.15)	−2.94 (−6.45 to 0.55)	.10
Diastolic blood pressure, mm Hg	1.92 (−0.25 to 4.09)	−0.24 (−2.38 to 1.90)	−2.16 (−4.62 to 0.30)	.08	2.44 (0.11 to 4.76)	0.49 (−1.81 to 2.78)	−1.95 (−4.9 to 1.00)	.19
Addi-QoL score	0.70 (−1.14 to 2.54)	1.74 (−0.10 to 3.57)	1.03 (−0.89 to 2.95)	.28	0.95 (−1.15 to 3.05)	0.37 (−1.73 to 2.47)	−0.58 (−3.48 to 2.32)	.69
SF-36 score								
Physical functioning	0.22 (−2.01 to 2.45)	2.77 (0.55 to 5.00)	2.55 (−0.52 to 5.63)	.10	−0.51 (−2.68 to 1.67)	1.66 (−0.51 to 3.82)	2.16 (−0.83 to 5.16)	.15
Role functioning–physical	−2.98 (−10.16 to 4.19)	4.78 (−2.39 to 11.96)	7.77 (−1.54 to 17.1)	.10	−5.89 (−13.9 to 2.15)	−3.40 (−11.4 to 4.65)	2.49 (−6.8 to 11.8)	.59
Role functioning–emotional	−0.02 (−4.45 to 4.42)	1.26 (−3.17 to 5.69)	1.28 (−4.84 to 7.39)	.68	0.99 (−5.34 to 7.31)	−1.11 (−7.43 to 5.21)	−2.1 (−5.44 to 1.25)	.21
Energy-fatigue	−0.31 (−4.34 to 3.11)	1.57 (−2.15 to 5.30)	2.18 (−2.56 to 6.93)	.36	−2.23 (7.20 to 2.74)	−2.68 (−7.66 to 2.29)	−0.46 (−5.97 to 5.06)	.87
Emotional well-being	−1.02 (−4.04 to 2.00)	2.07 (−0.94 to 5.07)	3.09 (−0.98 to 7.16)	.13	−0.91 (−3.69 to 1.87)	0.87 (−1.9 to 3.64)	1.78 (−1.41 to 4.97)	.27
Social functioning	−1.87 (−6.66 to 2.92)	3.11 (−1.63 to 7.85)	4.98 (−0.31 to 10.3)	.06	−2.25 (−7.62 to 3.12)	−0.60 (−5.91 to 4.71)	1.65 (−4.99 to 8.29)	.62
Pain	−1.58 (−5.60 to 2.45)	−0.17 (−4.19 to 3.84)	1.40 (−2.76 to 5.57)	.50	−0.30 (−5.21 to 4.61)	−0.22 (−5.12 to 4.68)	0.08 (−5.51 to 5.67)	.98
General health	0.23 (−2.92 to 3.37)	3.29 (0.15 to 6.43)	3.06 (−1.2 to 7.33)	.15	1.30 (−2.53 to 5.12)	0.80 (−3.02 to 4.62)	−0.49 (−5.76 to 4.78)	.85
Health change	2.51 (−2.19 to 7.20)	−2.00 (−6.69 to 2.69)	−4.5 (9.35 to 0.35)	.07	−3.90 (−9.13 to 1.32)	1.66 (−3.56 to 6.89)	5.56 (−1.65 to 12.8)	.13

Abbreviations: Addi-QoL, Addison’s Disease-Specific Quality of Life Questionnaire; BMI, body mass index (calculated as weight in kilograms divided by the square of height in meters); HOMA β, Homeostasis Model Assessment of β-Cell Function; HOMA-IR, Homeostatic Model Assessment for Insulin Resistance; SF-36, 36-Item Short Form Health Survey.

SI conversion factors: To convert alkaline phosphatase to microkatals per liter, multiply by 0.0167; calcium to millimoles per liter, multiply by 0.25; C-peptide to nanomoles per liter, multiply by 0.331; fructosamine to millimoles per liter, multiply by 5.581; glucose to millimoles per liter, multiply by 0.0555; insulin to picomoles per liter, multiply by 6.945; osteocalcin to micrograms per liter, multiply by 1; parathyroid hormone to nanograms per liter, multiply by 1; and vitamin D to nanomoles per liter, multiply by 2.496.

^a^ Treatment effects estimated using repeated-measures linear mixed model at days 30 and 120 for hydrocortisone and prednisolone, with treatment differences. Treatment effects are represented as estimated marginal mean (95% CI).

**Figure 2.**
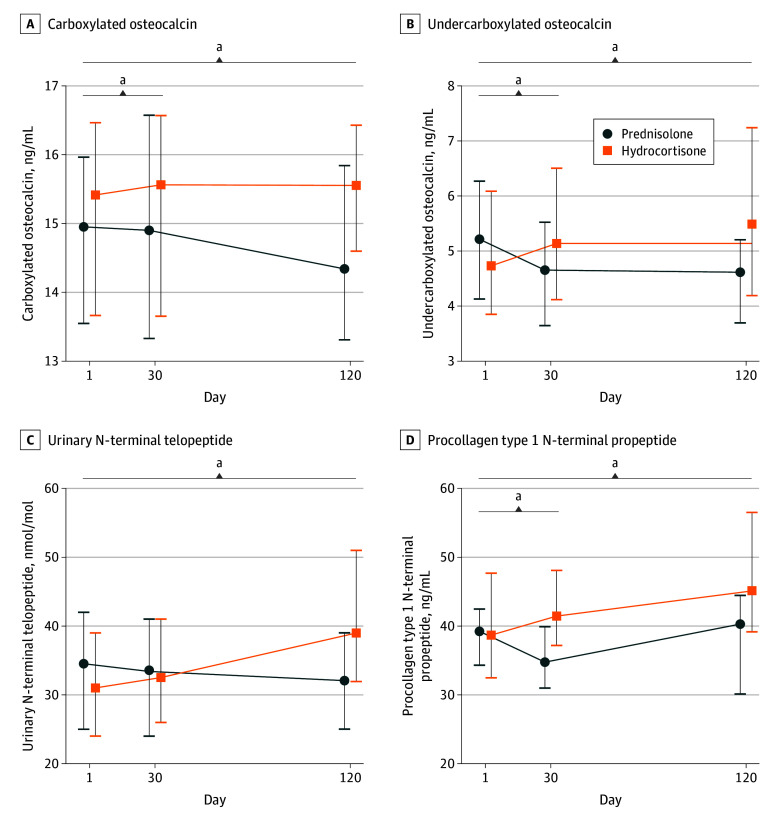
Line Graph of Bone Marker Profiles Line graph showing the trends of biochemical bone markers in the study. All data shown as medians with 95% CIs (error bars). SI conversion factor: To convert osteocalcin to micrograms per liter, multiply by 1. ^a^Significant interaction for treatment effect (*P* < .05).

**Figure 3.**
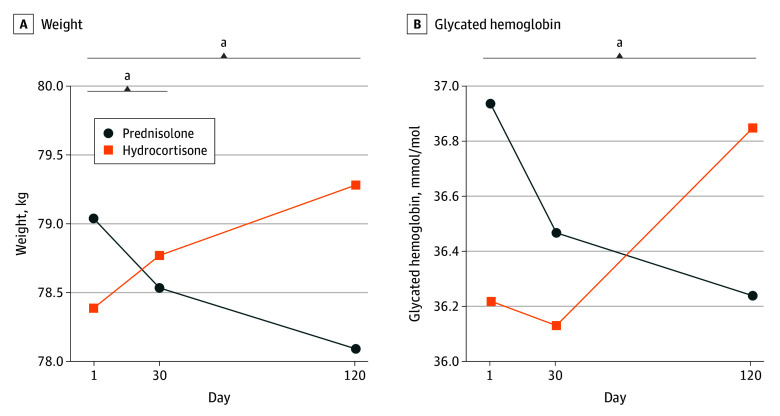
Line Graph of Cardiovascular Risk Marker Profiles Panels showing the trends of weight and HbA_1c_ in the study. All data are shown as mean. ^a^Significant interaction for treatment effect (*P* < .05).

Bone turnover was significantly slowed with prednisolone compared with hydrocortisone as evidenced by a significantly lower level of multiple bone markers, including carboxylated osteocalcin (mean treatment difference, −1.22 ng/mL; 95% CI, −2.35 to −0.10 ng/mL; *P* = .04), undercarboxylated osteocalcin (mean treatment difference, −1.38 ng/mL; 95% CI, −2.32 to −0.44 ng/mL; *P* = .005), urinary N-terminal telopeptide (mean treatment difference, −9.34 nmol/mmol; 95% CI, −15.40 to −3.29 nmol/mmol; *P* = .002), and procollagen type 1 N-terminal propeptide (mean treatment difference, −13.80 ng/mL; 95% CI, −22.20 to −5.49 ng/mL; *P* < .001) (Table 2 and Figure 2). The mean treatment group difference in weight reduction from baseline was −1.87 kg (95% CI, −3.02 to −0.72 kg; *P* = .002) for prednisolone treatment compared with hydrocortisone, and this was associated with concordant significantly greater reductions in body mass index (BMI; calculated as weight in kilograms divided by the square of height in meters; treatment difference, −0.52; 95% CI, −1.01 to −0.04; *P* = .04), waist circumference (treatment difference, −2.26 cm; 95% CI, −3.97 to −0.56 cm; *P* = .01), and HbA_1c _(treatment difference, −0.12% [−1.23 mmol/mol; 95% CI, −1.95 to −0.51 mmol/mol]; *P* = .001) (Figure 3). Fructosamine, fasting glucose, insulin, C-peptide, and Homeostatic Model Assessment for Insulin Resistance (HOMA-IR) were not significantly different between the treatments. There was no significant difference in lipids or blood pressure between the 2 arms (eTable 4 in Supplement 2 and Table 2).

Type of adrenal insufficiency (primary vs secondary) had no effect on all but 3 outcome measures. The notable expected exceptions were adrenocorticotropic hormone levels in which the 30-day effect size for secondary adrenal insufficiency was −153 ng/L (95% CI, 95% CI, −246 to −61 ng/L; *P* = .002) and bicarbonate at 30 days, with an effect size of 1.1 mEq/L (95% CI, 0.1-2.1 mEq/L) (to convert to millimoles per liter, multiply by 1) (*P* = .05) for secondary adrenal insufficiency. The adrenocorticotropic hormone interaction is related to the etiology, whereas bicarbonate reflects fludrocortisone use. Alanine aminotransferase showed significant interactions at 30 and 120 days, with effect sizes for secondary adrenal insufficiency of −6.5 U/L (95% CI, −11.6 to −1.4 U/L; *P* = .02) and −4.5 U/L (95% CI, −8.6 to −0.3 U/L; *P* = .04), respectively.

There was no difference in subjective health outcomes between hydrocortisone and prednisolone therapy (Table 2). Day 120 Addi-QoL data showed a 0.95 increase in score from baseline associated with hydrocortisone and a 0.37 increase with prednisolone (*P* = .69). There was no difference between treatments in any of the SF-36 domains, including physical functioning. energy fatigue, and general health.

There were 133 adverse events seen in 37 participants. Of these, 3 events were classed as serious, including an episode of acute gastroenteritis secondary to food poisoning in 1 participant and viral gastroenteritis in another participant. Only 1 participant was withdrawn; this individual was in the hydrocortisone-first arm and was found incidentally to have hyponatremia on blood testing at a routine outpatient appointment. This hyponatremia occurred while the patient was in the prednisolone period before the day 30 study visit (Figure 1). All 3 participants were treated with intravenous hydrocortisone. There were an additional 17 episodes of doubling glucocorticoid dose without an associated adverse effect in 9 participants for subjective perceived stress. There were no adrenal crises observed during the study.

There was no difference in the frequency of adverse events in one treatment vs the other. All adverse events are listed in eTable 5 in Supplement 2. The most common adverse events were viral illnesses (n = 34), lethargy (n = 12), and COVID-19 infection (n = 8).

## Discussion

To our knowledge, this is the first double-blind randomized clinical trial comparing low-dose prednisolone (2-5 mg) with conventional multiple-dose hydrocortisone for glucocorticoid replacement in adrenal insufficiency. Participants demonstrated greater reductions from baseline in weight, BMI, waist circumference, and HbA_1c_ level on day 120 of the prednisolone period compared with day 120 of the hydrocortisone period. Both regimens did not differ in safety as assessed by adverse events and in quality of life as assessed by validated subjective health questionnaires. Once-daily prednisolone replacement therapy is associated with improvements in cardiovascular and glycemic outcomes when compared with conventional hydrocortisone therapy, and our data support the conduct of long-term trials exploring the comparative cardiometabolic and other clinical impact of these alternative glucocorticoid replacement regimens. The potential mechanism of once-daily prednisolone causing less weight gain than multiple doses of hydrocortisone includes a lower steroid exposure or the possibility that once daily therapy has different effects on the circadian rhythm to multiple daily doses.^22^

Similar results have been seen in research evaluating the use of once-daily dual-release hydrocortisone, which is formulated to release a proportion of hydrocortisone immediately and the rest as extended release.^23^ Prednisolone shares a similar plasma profile to once-daily dual-release hydrocortisone, is rapidly absorbed, and has a half-life suitable for once-daily administration.^4,18,24^ Studies evaluating the use of once-daily dual-release hydrocortisone report reductions in weight, BMI, waist circumference, and HbA_1c_ level compared with conventional hydrocortisone or cortisone acetate glucocorticoid replacement.^25,26^ We demonstrate a significant treatment difference between prednisolone and hydrocortisone, with a mean −1.87-kg reduction in weight. In those treated with the once-daily hydrocortisone formulation in the DREAM trial, there was a mean reduction of −2.1 kg.^25^ Similar concordant treatment differences with prednisolone were seen with a reduction in waist circumference of 2.26 cm compared with a reduction of 2.5 cm seen with the once-daily hydro cortisone formulation. In a 10-year, prospective randomized clinical trial^2^ comparing once-daily dual-release hydrocortisone with conventional glucocorticoid replacement, patients receiving once-daily dual-release hydrocortisone had a reduction in weight from 68.8 to 66.1 kg compared with an increase in weight from 67.1 to 72.5 kg in those receiving conventional glucocorticoid replacement therapy with twice- or thrice-daily cortisone acetate or standard hydrocortisone. A study of 10 individuals who switched from standard hydrocortisone regimens to once-daily dual-release hydrocortisone showed a mean reduction of fat mass by 3.2 kg.^27^ Taken together, these findings suggest that the metabolic benefits seen with once-daily dual-release hydrocortisone and prednisolone may be due to avoiding glucocorticoid exposure in the evening.^28,29^ It is also possible that the longer half-life of prednisolone may in turn lead to an overall lower exposure to glucocorticoid compared with multiple doses of any glucocorticoid.^4^

Prednisolone binding to the glucocorticoid receptor is 2.26 times more avid than cortisol.^30^ The longer half-life of prednisolone may be due to slower metabolism caused by the double bond between C1 and C2. The combination of increased avidity and the longer half-life may explain its increased potency, with 3 mg being approximately similar to 20 mg of hydrocortisone in divided doses.

Osteocalcin and procollagen type 1 N-terminal propeptide are associated with bone formation,^31,32^ whereas urinary N-terminal telopeptide and serum C-terminal telopeptide are associated with bone resorption.^33^ In this study, 4 months of prednisolone treatment led to significant reductions in all 4 markers compared with conventional hydrocortisone. Future prospective studies with longer follow-up of a larger number of participants are needed to demonstrate clinically meaningful changes in bone mineral density or incidence of fractures with long-term use of prednisolone.

Our study did not detect any differences in quality of life between either treatment. This finding is in keeping with a previous double-blind study comparing 20 mg of hydrocortisone and 5 mg of prednisolone in patients fasting for 1 month.^34^ The study used the Addi-QoL but is confounded by each treatment period lasting 14 days.

### Limitations

This study has some limitations. The major limitation is the use of biomarkers as opposed to event outcome data. Use of surrogate markers of cardiovascular risk or bone health may correlate with myocardial infarctions, revascularization procedures, and fractures but does not provide the same level of evidence. As the first head-to-head comparison, this study had to look at short-term outcomes, being limited by time. Additional studies will likely look at medium-term outcomes, such as bone mineralization from dual-energy X-ray absorptiometry, after which cohort studies will be required to quantify major adverse cardiac events, comorbidity, and fracture data to ascertain which glucocorticoid regimen is superior.

## Conclusions

This randomized clinical trial found slower bone turnover as well as potential benefits for important cardiovascular and glycemic markers with once-daily low-dose prednisolone treatment compared with multiple-dose hydrocortisone. Low-dose prednisolone is a safe once-daily treatment that can be used for glucocorticoid replacement in adrenal insufficiency.

## Notes

Supplement 1.
Trial Protocol and Statistical Analysis Plan

Supplement 2. **eMethods.** Supplementary methods **eTable 1.** Assay platform and performance specification for analytes measured at North West London Pathology (NWLP) **eTable 2. **Aetiology of adrenal insufficiency in study participants **eTable 3. **Frequency of steroid regimens used during the main study **eTable 4. **Extended outcome data of all secondary outcomes **eTable 5. **Tabulation of all adverse events according to concurrent glucocorticoid at the time

Supplement 3.
Data Sharing Statement
