# Supplementary material for: Prednisolone Once Daily vs Hydrocortisone Thrice Daily in Hypoadrenalism: A Randomized Clinical Trial
Source: JAMA Netw Open. 2026 Mar 24;9(3):e262982. doi: 10.1001/jamanetworkopen.2026.2982 (PMC13014172; doi:10.1001/jamanetworkopen.2026.2982)
Supplement: Supplement 1. — Trial Protocol and Statistical Analysis Plan [file jamanetwopen-e262982-s001.pdf]

PRED-AID Study

Safety and Efficacy of Prednisolone in Adrenal Insufficiency Disease (PRED-AID Study)

Version 2.0
30th September 2022

SPONSOR: Imperial College London

FUNDERS: National Institute for Health Research (NIHR)
Imperial Health Charity

STUDY COORDINATION CENTRE: Section of Investigative Medicine
Imperial College London
6th Floor Commonwealth Building
Du Cane Road
London
W12 0NN

IRAS Project ID: 201045
REC reference: 19/LO/0083
EudraCT reference: 2018-001502-28

Protocol authorised by:

Professor Karim Meeran (Chief Investigator)

Signature: Karim Meeran. **Date:30th September 2022**

Study Management Group

Chief Investigator:	Prof Karim Meeran, Section of Investigative Medicine, Imperial College London
Co-investigator:	Dr Sirazum Choudhury, Section of Investigative Medicine, Imperial College London
Co-investigator:	Prof Tricia Tan, Section of Investigative Medicine, Imperial College London
Co-applicant:	Prof Toby Prevost, Imperial Clinical Trials Unit
Statistician:	Dr Suzie Cro, Imperial Clinical Trials Unit
Study Administrator:	Mr Nima Khandan-Nia, Section of Investigative Medicine, Imperial College London
Trial Management:	Dr Sirazum Choudhury, Section of Investigative Medicine, Imperial College London

Study Coordination Centre

For general queries, supply of trial documentation, and collection of data, please contact:

Study Coordinator: Dr Sirazum Choudhury
Address: Section of Investigative Medicine
Imperial College London
6th Floor Commonwealth Building
Du Cane Road
London
W12 0NN
Tel: 020 3313 3052
Email: steroids@imperial.ac.uk

Clinical Queries

Clinical queries should be directed to Dr Sirazum Choudhury who will direct the query to the appropriate person

Sponsor

Imperial College London the main research Sponsor for this study. For further information regarding the sponsorship conditions, please contact the Head of Regulatory Compliance at:

Research Governance and Integrity Team (RGIT)
Imperial College London and Imperial College Healthcare NHS Trust
Room 215, Level 2, Medical School Building

Norfolk Place
London, W2 1PG
Tel: 0207 594 1862
Imperial College - Research Governance and Integrity Team (RGIT) Website

Funder

This study is funded by the NIHR and the Imperial Health Charity

National Institute for Health Research
Leeds Office
21 Queen Street
Leeds, LS1 2TW
Tel: 0113 343 2314

Imperial Health Charity
Second Floor
178-180 Edgware Road
London W2 2DS
Tel: 020 3857 9840

This protocol describes the PRED-AID study and provides information about procedures for entering participants. The protocol should not be used as a guide for the treatment of other participants; every care was taken in its drafting, but corrections or amendments may be necessary. These will be circulated to investigators in the study, but centres entering participants for the first time are advised to contact the trials centre to confirm they have the most recent version. Problems relating to this trial should be referred, in the first instance, to the study coordination centre.

This trial will adhere to the principles outlined in the Medicines for Human Use (Clinical Trials) Regulations 2004 (SI 2004/1031), amended regulations (SI 2006/1928) and the International Conference on Harmonisation Good Clinical Practice (ICH GCP) guidelines. It will be conducted in compliance with the protocol, the Data Protection Act and other regulatory requirements as appropriate.

Table of Contents

1. INTRODUCTION
1.1 BACKGROUND
2. STUDY OBJECTIVES
3. STUDY DESIGN
3.1 STUDY OUTCOME MEASURES
4. PARTICIPANT ENTRY
4.1 PRE-RANDOMISATION EVALUATIONS
4.2 INCLUSION CRITERIA
4.3 EXCLUSION CRITERIA
4.4 WITHDRAWAL CRITERIA
5. RANDOMISATION AND ENROLMENT PROCEDURE
5.1 RANDOMISATION OR REGISTRATION PRACTICALITIES
5.2 UNBLINDING
6. TREATMENTS
6.1 TREATMENT ARMS
6.2 DOSE MODIFICATIONS FOR TOXICITY
6.3 PREMEDICATION
6.4 INTERACTION WITH OTHER DRUGS
6.5 DISPENSING AND ACCOUNTABILITY
6.6 COVID-19 CONCURRENT INFECTION MANAGEMENT PLAN
7. PHARMACOVIGILANCE
7.1 DEFINITIONS
7.2 CAUSALITY
7.3 REPORTING PROCEDURES
7.4 REFERENCE SAFETY INFORMATION FOR HYDROCORTISONE
7.5 REFERENCE SAFETY INFORMATION FOR PREDNISOLONE
8. ASSESSMENT AND FOLLOW-UP
8.1 LOSS TO FOLLOW-UP
8.2 TRIAL CLOSURE
9. STATISTICS AND DATA ANALYSIS
10. MONITORING
10.1 RISK ASSESSMENT
10.2 MONITORING AT STUDY COORDINATION CENTRE
11. REGULATORY ISSUES
11.1 CTA
11.2 ETHICS APPROVAL
11.3 CONSENT
11.4 CONFIDENTIALITY
11.5 INDEMNITY
11.6 SPONSOR
11.7 FUNDING
11.8 AUDITS AND INSPECTIONS
12. TRIAL MANAGEMENT
13. PUBLICATION POLICY
14. REFERENCES

APPENDICES
APPENDIX 1. Schematic of pre-randomisation events
APPENDIX 2. Schematic of study periods and visits

GLOSSARY OF ABBREVIATIONS

**ACTH**	Adrenocorticorticotropic hormone- pituitary hormone which modulates cortisol
**Addi-QoL**	Addison's disease specific Quality of Life Questionnaire- a subjective health questionnaire validated in adrenal insufficiency
**AE**	Adverse event
**AI**	Adrenal insufficiency- Condition in which individuals are unable to synthesise steroid hormones such as cortisol, a stress hormone. Without treatment, this condition is fatal.
**AR**	Adverse Reaction
**BALP**	Bone specific Alkaline Phosphatase- a bone formation marker
**BMI**	Body Mass Index
**BNP**	Brain Natriuretic Peptide a cardiac marker
**CBG**	Cortisol Binding Globulin- a protein which binds cortisol in the blood
**CK**	Creatine Kinase- a muscle enzyme used to indicate muscle damage
**ECG**	Electrocardiogram- tracing of the heart's electrical activity
**FBC**	Full Blood Count
**GNCQ**	German National Cohort Questionnaire- a measurement tool for infection rates
**HbA1c**	Haemoglobin A1c- a diabetic marker
**HOMA-IR**	Homeostatic Model Assessment for Insulin Resistance- an indicator of insulin sensitivity
**hs-CRP**	High sensitivity C-reactive Protein a cardiovascular risk marker
**ICHNT**	Imperial College Healthcare NHS Trust- Incorporates St Mary's Hospital, Hammersmith Hospital and Charing Cross Hospital
**ICTU**	Imperial Clinical Trials Unit
**IMP**	Investigational Medicinal Product
**LFT**	Liver Function Tests
**NIHR**	National Institute of Health Research
**NTX**	N-Telopeptide Crosslinks- a bone reabsorption marker
**OC**	Osteocalcin- a bone formation marker
**P1NP**	Type 1 Pro-collagen N-terminal peptide- a bone formation marker
**PTH**	Parathyroid Hormone
**SAE**	Serious Adverse Event
**SD**	Standard Deviation
**SF-36**	Short Form Health Survey-36- a subjective health questionnaire
**SmPC**	Summary of Product Characteristics
**SUSAR**	Suspected Unexpected Adverse Reaction
**TFT**	Thyroid Function tests

KEYWORDS

Addison's disease, adrenal insufficiency, corticosteroids, glucocorticoids, hydrocortisone, hypoadrenalism, hypopituitarism, prednisolone.

STUDY SUMMARY

TITLE: Safety and Efficacy of Prednisolone in Adrenal Insufficiency Disease (PRED-AID Study)

DESIGN: Phase 3, two-arm, two-period, double-blind crossover study

AIMS: To compare the effects of two routine treatments of adrenal insufficiency (AI), low dose prednisolone and standard regimens of hydrocortisone.

OUTCOME MEASURES: The effects of both treatment regimens on the following will be compared:

- Bone turnover
- Cardiovascular risk
- Glycaemic control
- Infection rates and severity
- Immunology profiles
- Safety
- Wellbeing
- Compliance

POPULATION: Patients with stably treated primary or secondary hypoadrenalism who are attending endocrinology clinics in the UK

ELIGIBILITY: Patients able to give informed consent, between 18 and 70, who have been diagnosed with AI for over 6 months and have remained on a stable hormone replacement regimen for at least 3 months.

TREATMENT AND DURATION: Either:

a) Four months of prednisolone once daily followed by 4 months of hydrocortisone daily (once daily or multiple doses daily) or;
b) Four months of hydrocortisone daily (once daily or multiple doses daily) followed by 4 months of prednisolone once daily

The dose of the participant's usual regimen will be unchanged from their pre-study dose. The dose of the alternative medication to be used will be elucidated prior to each participant's enrolment on the study.

1. INTRODUCTION

1.1 BACKGROUND

There are approximately 27,000 individuals with Adrenal Insufficiency (AI) in the UK with an annual incidence 3140 new cases (1,2). AI is caused either by primary adrenal disease or is secondary to pituitary or hypothalamic disease. Untreated, the 2-year mortality is 85%(3). AI is treated by replacing endogenous steroids using oral glucocorticoids(4), with the goal of mimicking the physiological diurnal cortisol rhythm. Although life expectancy has improved since the introduction of treatment(5), there is still increased mortality with AI, associated with over-replacement(5,6). Over-replacement increases the long-term risks of malignancy, diabetes, cardiovascular disease and osteoporosis(5,7). If under-replaced, patients experience symptoms such as lethargy, depression, nausea and are exposed to the risk of life-threatening Addisonian crises(8). It is important to strike the correct balance with steroid replacement to optimize quality of life and prevent acute crises but without increasing the long-term risk of mortality and morbidity.

The most commonly prescribed therapy is hydrocortisone. With a short half-life of 2 hours(9), hydrocortisone requires most commonly thrice-daily administration, which reduces patient compliance. Moreover, hydrocortisone is expensive at £84.44 per month for 20mg daily (10mg in the morning, 5mg at noon and 5mg in the evening).

Prednisolone is an alternative to hydrocortisone that is structurally similar to standard hydrocortisone but has a longer half-life (2-4 hours), permitting it to be given once-daily. Prednisolone is recommended as an alternative in the Endocrine Society's Clinical Practice Guidelines(10). In addition to the benefit of once-daily administration, prednisolone is 35-60 times cheaper than hydrocortisone at £3.21-5.35 per month.

In the past, prednisolone was used for replacement at a dose of 7.5 mg daily, in a one-size-fits-all fashion, and this practice was associated with the development of osteoporosis and diabetes, i.e. the dose was too high (11-14). However, two developments make low-dose prednisolone a viable choice for replacement in AI: (1) new evidence that the appropriate dose is much lower (2-5mg) (15); (2) a new assay developed at Imperial College Healthcare NHS Trust, used to identify patients as slow versus fast metabolisers, allowing us to customise doses to individual patients.

There is currently a paucity of evidence supporting the use of prednisolone at lower doses (5mg or less) in AI, with the exception of a few case reports of AI patients doing well on doses of 3mg (16). There have been no prospective studies comparing low-dose prednisolone to established hydrocortisone therapy assessing patient satisfaction, bone or glycaemic outcomes. This study aims to address this dearth of evidence, by investigating the equivalence of prednisolone to standard hydrocortisone in replacing the steroid requirements of patients with AI.

1.2 RATIONALE FOR CURRENT STUDY

This study will investigate the equivalence of low-dose prednisolone and standard hydrocortisone regimens in their effect on biochemical, anthropometric, immunological and subjective health outcomes.

In contrast to past data based on excessive doses of prednisolone used in steroid replacement therapy, it is expected that low-dose prednisolone therapy will have demonstrate equivalent outcomes to standard hydrocortisone.

There are no benefits to participants who are taking part in this study. There will be additional burdens on participants including a pre-enrolment dose-finding period. During this time, which will be a minimum of 2 weeks, participants will be assessed on a test dose of the alternative steroid replacement therapy to their baseline therapy. For example, a patient usually on prednisolone will be trialled on hydrocortisone. During this time, their regimen will be titrated according to the standard practices used at Imperial College Healthcare NHS Trust (ICHNT). This may involve blood tests during the dose-finding period.

Prior to each study visit, participants will be asked to observe an overnight fast. This is required to ensure good quality data, in particular with fasting glucose levels and fasting lipid levels. Participants accustomed to once daily prednisolone will have to take extra tablets for a three-times daily regimen, which will be necessary to preserve the double-blinded design.

Participants will receive prednisolone for steroid replacement. Previous studies have highlighted deleterious effects on bone and glycaemic handling associated with long term use of high dose prednisolone, although this is not expected with low-dose prednisolone. Other steroid related adverse effects such as myopathy will be screened for during the reporting of symptoms and using creatine kinase measurements. However as low dose steroid doses are used only in this trial, the above risks are minimal. Nevertheless, bone markers will be measured on study days as part of the trial.

As participants will be changing their steroid treatment as part of this study, there is a risk of undertreatment. This risk will be minimised as patients will be trialled on the new regimen if they have not previously received it before. Possible symptoms will include tiredness, dizziness, pre-syncope, syncope, palpitations, and abdominal pain. These will be detected through symptom reporting, which will take place during all visits and telephone consultations. Participants will also have a 24-hour emergency contact number for a study clinician which they will be instructed to call if they feel unwell.

At times of intercurrent illness, participants will be asked to double the tablets that they are taking. This is usual clinical practice and will be overseen by a study clinician, who will be available on a 24-hour emergency contact number. Specifically, participants will be asked to take an additional tablet from their morning bottle for the first double dose, before doubling the number of tablets that they usually take from each bottle thereafter. Therefore, instead of taking one tablet from the morning, noon and afternoon bottles, participants will take two from each bottle at the allocated time after the special arrangement for the first double dose.

The only procedure being undertaken as part of this study is venepuncture, thus there may be slight pain when this is performed. Efforts will be made to minimise the frequency and quantity of blood taken

2. STUDY OBJECTIVES

To compare the effects of two routine treatments of AI, low dose prednisolone and standard regimens of hydrocortisone, on indicators of:

- Bone turnover
- Cardiovascular risk
- Glycaemic control
- Infection rates and severity
- Immunology profiles
- Safety
- Wellbeing
- Compliance

3. STUDY DESIGN

Patients with stably treated primary or secondary hypoadrenalism on either hydrocortisone or prednisolone replacement will be recruited to this study from patients attending endocrinology clinics in the UK.

This study will be a phase 3, two-period, randomised cross-over, double-blind study with two treatment arms:

A) Participants given once daily low dose prednisolone (at 2-5 mg, determined by the serum level of prednisolone at 8 hours after a test dose) with placebo tablets at lunch and in the afternoon for four months (first period); followed by hydrocortisone once daily, or multiple doses daily (at their stable therapeutic regimen or the dose identified post-screening) for four months in the second period

B) Participants given hydrocortisone once times daily, or multiple doses daily (at their stable therapeutic regimen or the dose identified post-screening) for four months (first period); followed by given once daily low dose prednisolone (at 2-5 mg, determined by the serum level of prednisolone at 8 hours after a test dose) with placebo tablets at lunch and in the afternoon for four months in the second period

Participants will be randomly assigned to begin with either treatment arm. Each treatment period will be continued for 4 consecutive months. Participants will then undergo a washout period during which they will be returned to their baseline treatment for a minimum period of 2 weeks, before starting on the alternate treatment arm. The aim is to assess the effect of each treatment arm on bone health, surrogate markers of cardiovascular risk, glycaemic control, infection rates, immunology profiles, patient wellbeing and compliance.

Participant randomisation will be blocked to ensure that equal numbers of patients receive the treatments in the two orders. Each block will be randomised according the type of AI (primary vs secondary).

Sample size

Fifty-five patients will be recruited, allowing for a 20% drop-out rate. With 44 completers analysed there is a minimum of 90% power to detect a 1.5 µg/L effect in osteocalcin with a two-sided test at the 5% level of significance, assuming an estimate of the SD at a single timepoint of SD of 3.0 (14). This assumes cautiously that the correlation between the repeated

measurements within patients across the two periods is 0.5, whereas a previous crossover trial(17) showed a correlation of 0.8.

Following a pilot study to elucidate a reference range for osteocalcin in healthy volunteers and patients with AI, it has been demonstrated that individuals can have reductions of up to 2.4 µg/L in the short term after changes in their steroid replacement regimen. It is anticipated that that larger changes in osteocalcin will be seen when patients continue on their new regimens for a longer duration.

3.1 STUDY OUTCOME MEASURES

Primary Outcome:

- Bone health
    - assessed by measurement of change in osteocalcin, a bone formation marker

Secondary Outcomes:

- Other markers of bone health
    - assessed by measurement of change in additional bone markers and bone profile including procollagen type-1 N-terminal propeptide (P1NP), bone specific alkaline phosphatase (BALP), corrected calcium, parathyroid hormone (PTH), vitamin D and urinary N-terminal telopeptide (NTX).

- Surrogate markers and risk factors for cardiovascular disease
    - including anthropometric markers such as: blood pressure, heart rate, BMI (height on first occasion), weight and waist-hip circumference ratio.
    - cardiovascular risk assessed by measurement of high-sensitivity CRP, high-sensitivity troponin I and BNP.

- Glycaemic control
    - assessed by HbA1c, fructosamine, fasting glucose levels and insulin resistance represented by HOMA-IR

- Infection rates and severity
    - assessed by completion of the German National Cohort Questionnaire (GNCQ)

- Immunology profiles
    - Assessed by measurement and assessment of soluble immunological analytes and isolated white cell populations.

- Safety
    - assessed by reporting of symptoms of steroid deficiency and myopathy and review of routine monitoring blood tests including full blood count (FBC), renal profile, liver function tests (LFTs), creatine kinase (CK), Adrenocorticotropic hormone (ACTH) cortisol binding globulin (CBG) and bicarbonate.

- Wellbeing
    - assessed by subjective health questionnaires including the SF-36 (18) and Addi-QoL (19)

- Compliance to regimen
    - assessed by collecting the remaining unused tablets at the end of each treatment arm

4. PARTICIPANT ENTRY

4.1 PRE-RANDOMISATION EVALUATIONS (APPENDIX 1)

All volunteers will attend a screening visit prior to commencing the study. At the screening visit, each volunteer will be assessed by a study clinician, counselled and informed consent obtained. Their suitability for the study will be evaluated with a full medical history, physical examination, observations (height, weight, blood pressure, heart rate, waist circumference and hip circumference), and urinalysis (and pregnancy test for females of child bearing potential). To prevent unnecessarily performing invasive tests or consuming the volunteer's time, the subsequent events will only be performed if the patient is still potentially eligible for the study and has not met any of the exclusion criteria to this point.

Blood tests will be performed including fasting renal, bone and lipid profiles, bicarbonate, full blood count (FBC), glucose, insulin, fructosamine, HbA1c, creatine kinase(CK), Adrenocorticotropic Hormone (ACTH), cortisol binding globulin (CBG), parathyroid hormone (PTH), vitamin D, bone-specific alkaline phosphatase (BALP), osteocalcin (OC), procollagentype1 N-terminal propeptide(P1NP); hs-CRP, hs-Troponin I, BNP, assessment of soluble immunological antigens and assessment of white cell populations. The previously collected urine sample will also be kept for quantification of N-terminal crosslinks (NTX). The volunteer will also be asked to complete three questionnaires, the Addison's Disease specific quality of life questionnaire (Addi-QoL), the short form health survey-36 (SF36) and the German National Cohort Questionnaire (GNCQ).

Females of child bearing potential who are suitable for the study will be asked to maintain adequate contraception for the duration of the study. Other than the combined oral contraceptive pill, all other types of contraception will be acceptable.

If the volunteer is determined to be suitable, their daily prednisolone replacement dose will be assessed to take forward into the study if it has not previously been done. This will be completed in accordance with the standard practice at ICHNT which involves administration of fixed dose of prednisolone (3mg or 4mg, determined clinically) followed by an 8-hour blood test to measure prednisolone concentration. Depending on the 8-hour level and the clinical picture, the prednisolone dose is titrated up or down and repeat 8-hour levels are performed. This is done until the correct replacement dose produces an 8-hour prednisolone level which is within the target range. Volunteers will be trialled on their replacement dose of prednisolone for at least 2 weeks prior to randomisation on the study to ensure safety. We anticipate that the majority of participants will require 2-5 mg. This phase will be overseen by a clinician experienced in titrating prednisolone doses and will be guided by the clinical picture. Any deviation from standard clinical care will be undertaken only under the direct guidance of the CI (himself a consultant endocrinologist experienced in managing these patients).

If a volunteer has not previously been established on hydrocortisone, they will be trialled on 10mg in the morning, 5 mg at lunch and 5 mg in the evening for a period of at least 2 weeks, prior to randomisation to the study. Their tolerance of this dose will be assessed and titrated where necessary to ascertain the regimen to be taken forward into the study. The dose titration

will be done in accordance with the standard practice at ICHNT. This phase will be overseen by a clinician experienced in titrating hydrocortisone doses and will be guided by the clinical picture. Any deviation from standard clinical care will be undertaken only under the direct guidance of the CI (himself a consultant endocrinologist experienced in managing these patients).

Vitamin D levels can influence the measured levels of bone markers and will be assessed at screening. Individuals with low levels (<40 nmol/L in most participants) may receive vitamin D replacement and may have to continue with vitamin D supplementation for the duration of the study on a fixed regimen. This will be decided on a case by case basis by the Chief Investigator and is a part of standard care.

Participants with secondary adrenal insufficiency may require replacement of other hormones (e.g. thyroid hormone, testosterone or growth hormone). Individuals will need to be replaced with hormones at stable doses for 3 months prior to enrolling on the study.

4.2 INCLUSION CRITERIA

- Aged 18 – 70 years
- Male or female
- Diagnosed with AI for over 6 months according to standard diagnostic criteria
- Established on stable HC replacement or prednisolone replacement, dose not altered for at least 3 months
- Established on a stable dose of Fludrocortisone, if taking, dose not altered for at least 3 months
- Participants taking other hormone replacements (e.g. levothyroxine, testosterone or growth hormone in secondary adrenal insufficiency) are accepted providing that their replacement doses have not altered for at least 3 months
- Participants who are otherwise healthy enough to participate, as determined by pre-study medical history and physical examination.
- Participants who are able and willing to give written informed consent to participate in the study.

4.3 EXCLUSION CRITERIA

- Participants with a diagnosis of Type 1 or Type 2 diabetes mellitus.
- Unable to give informed consent.

- Taking supplements or herbal medications that the participant is unwilling or unable to stop prior to and during the study period e.g. St John's Wort (may decrease prednisolone levels), Cat's claw, Echinacea (immunomodulatory properties).

- Currently taking medications that alter CYP3A4 metabolism of glucocorticoids that the participant is unwilling or unable to stop prior to and during the study period e.g. phenytoin, phenobarbital, rifampicin, rifabutin, carbamazepine, primidone, aminoglutethimide, itraconazole, ketoconazole, ciclosporin or ritonavir.

- Pregnancy, taking the combined oral contraceptive pill, or oral oestrogen replacement therapy due to the effects on cortisol binding globulin levels and determination of prednisolone levels. Transdermal oestrogen replacement is permitted.

- Diagnosis of congenital adrenal hyperplasia, untreated

4.4 WITHDRAWAL CRITERIA

Participants will be free to withdraw from this study at any time without prejudicing any further treatment. Participants will also be withdrawn from the study if they lose the capacity to consent. If participants experience any significant AEs, they may be withdrawn following a review by the Chief Investigator.

Upon withdrawal, participants will be returned to their baseline steroid replacement therapy, which may be titrated by a study clinician if necessary. They will continue to be followed-up by their direct care team at their usual endocrinology outpatient clinic.

Following withdrawal, samples and data previously collected by study team will continue to be held and may be used in the data analysis phase. Participants will be informed of this at the time of obtaining consent.

It is understood by all concerned that an excessive rate of withdrawals can render the study un-interpretable; therefore, unnecessary withdrawal of patients should be avoided. Should a participant decide to withdraw from the trial, all efforts will be made to report the reason for withdrawal as thoroughly as possible and they will be encouraged to continue to provide follow-up data for the remaining trial visits for the current treatment period.

5. RANDOMISATION AND ENROLMENT PROCEDURE

5.1 RANDOMISATION OR REGISTRATION PRACTICALITIES

Randomisation will be managed through the Oracle InForm data capture system. This is a validated GCP-compliant data capture and management system. The allocation to study arm will be based on the original randomisation list populated and held by an independent statistician at the ICTU, which will be available for reference and back-up if the InForm database is unavailable.

Participant demographic data will be uploaded to the InForm system at enrolment, when they will be assigned a Study ID. Participants who successfully complete the two-week dose finding period (if required) will then be randomised to a study arm, receiving a Study Drug ID based on their blocking factor. The Imperial pharmacy will be made aware of the allocation and will dispense the appropriate blinded study medication and specified placebo at the patient's individual dose, for the relevant study period.

5.2 UNBLINDING

This is a double-blind study. The randomisation lists and/or code break envelopes will be held by:

1. Pharmacy at the ICHNT (randomisation spreadsheet)
2. Mr Nima Khandan-Nia - administrator in the department of Investigative Medicine
3. Dr Karen Mosley, General Manager, NIHR Clinical Research Facility, in a secure area within the centre
4. Mr Jacob Bonner, Quality Assurance and Governance Manager, NIHR Clinical Research Facility, in a secure area within the centre
5. Professor Tricia Tan- unblinded co-investigator

In the case of a medical emergency or in the event of a serious medical condition, when knowledge of treatment allocation is essential for the clinical management or welfare of the participant, an investigator or other physician managing the subject may decide to unblind that subject's treatment code. They should therefore request and obtain the relevant code-break envelope from Dr Karen Mosley or her designated deputy if she is away. Alternatively, Professor Tan can be contacted via the Imperial College Healthcare NHS Trust switchboard and will also be in possession of the relevant code-break envelope.

The investigator must sign and date the open unblinding envelope, as soon as is reasonably possible. Within 24 hours of the code break, the reason for the code break must be documented on the envelope. The Investigator will also record the date and reason for revealing the blinded treatment assignment for that subject in the CRF and in the subject's medical notes. The sponsor should also be notified.

6. TREATMENTS

6.1 TREATMENT ARMS

This is a two-arm, two-period crossover study. Participants will be randomly assigned to either arm (A) or arm (B), which will dictate the order in which the two alternative treatments are administered (Appendix 2):

A) Participants given once daily low dose prednisolone (at 2-5 mg, determined by the serum level of prednisolone at 8 hours after a test dose) with placebo tablets at lunch and in the afternoon for four months (first period); followed by hydrocortisone once daily or multiple doses daily (at their stable therapeutic regimen or the dose identified

post-screening, anticipated to be 10mg, 5mg, 5mg for most participants) for four months in the second period

B) Participants given hydrocortisone once daily or multiple doses daily (at their stable therapeutic regimen or the dose identified post-screening, anticipated to be 10mg, 5mg, 5mg for most participants) for four months (first period); followed by given once daily low dose prednisolone (at 2-5 mg, determined by the serum level of prednisolone at 8 hours after a test dose) with placebo tablets at lunch and in the afternoon for four months in the second period

Both study periods in each arm will last for 4 months. In-between study periods, there will be a minimum 2-week washout period, during which participants will return to their pre-study baseline treatment.

After a participant has completed the study periods, they will return to their baseline treatment.

All study specific medication will be administered via the oral route. To ensure the integrity of the double-blinded study design, all the medication and placebos will be bulk produced by Activase Pharmaceuticals Ltd, who will be providing the medications free of charge. The bulk material will be sent to the Tiofarma who will bottle and label the medication.

The medication will be dispensed by the ICHNT pharmacy who will be responsible for ensuring that the participants randomised to each study arm receive their study medication in the correct order corresponding to the study period.

It is anticipated that the study medication will be delivered to the ICHNT pharmacy in bulk and will be dispensed according to the need of each individual participant. The excess stock of tablets will be held by Tiofarma and will be delivered to ICHNT pharmacy if their stocks are running low.

6.2 DOSE MODIFICATIONS FOR TOXICITY

Dose modifications for toxicity will not be required in this study. Participants will already be accustomed to one of the two treatments in this study, as they will have been using the medication at the same dose for at least 3 months prior to enrolling onto the study.

If a participant is naïve to the alternative steroid medication, they will be given a trial of at least 2-weeks prior to randomisation. During this time, the dose of the alternative medication will be titrated, according to the standard clinical practices used at ICHNT.

6.3 PREMEDICATION

Vitamin D levels will be checked in each participant at screening. All participants will be required to demonstrate replete vitamin D levels (>40 nmol/L) during the trial as low vitamin D levels directly influence the concentration of bone markers such as osteocalcin. Participants with low vitamin D levels at screening (<40 nmol/L) are anticipated to receive a dose of 20,000 units for a period of 6 weeks before starting the study. Specific vitamin D replacement regimens may vary and some participants may have repeat vitamin D measurements but this will be assessed on a case by case basis, depending on the clinical picture. The final decision regarding vitamin D replacement regimens will lie with the Chief Investigator.

During the study all participants will be prescribed 800 – 1000 units daily for the duration of the trial, as is expected in standard care. Any deviation from this regimen will be decided upon on a case-by-case basis according to clinical requirement and agreed with the Chief Investigator.

6.4 INTERACTION WITH OTHER DRUGS

Participant require steroid replacement therapy to manage their medical condition for the rest of their lives. This should not impede them from taking other medications if indicated.

6.5 DISPENSING AND ACCOUNTABILITY

All the medication and placebos will be produced as bulk material by Activase Pharmaceuticals Ltd, who will be providing the medications free of charge. The bulk material will be sent to Tiofarma who will bottle and label the medication. QP release will be done by Tiofarma.

The medication doses required by each participant will be individual and determined according to the participant's previous experience with the medication, or else the outcome of the dose-finding period if the participant has not previously used one of the study medications. At randomisation, the participant will be given a Study Drug ID, allocated according to the blocking factor. The clinician will notify the pharmacy of the dose of hydrocortisone, and prednisolone needed by the participant and the sizes of placebo to be dispensed when the participant is on the prednisolone treatment period.

The medication will be dispensed by the ICHNT pharmacy who will be responsible for ensuring that the participants randomised to each study arm receive their study medication in the correct order corresponding to the study period, as dictated by their Study Drug ID, which will be cross-checked against the randomisation list.

At times of intercurrent illness, participants will have the autonomy to double their steroid dose. This is performed independently as part of their usual care. They will be asked to inform a study physician at any time they double their doses.

Individual patients have their own routines as to how they double their steroids. A common routine is for the participant to double their steroid dose for a minimum of three days, or else as long as the period of intercurrent illness. After the illness has resolved, the participant will usually reduce their steroid dose back to the usual regimen.

In practice during this study, participants will double their steroid dose by taking double the tablets that they usually administer from each bottle. Therefore if a participant takes one tablet from the morning bottle, one from the noon bottle, and one from the evening bottle, they will instead take two from each bottle. The only exception to this will be when they take their first dose of doubled steroids.

For the first doubled dose, participants will be asked to take their usual tablet, but to double their first dose with a tablet from the morning bottle. This is necessary as participants who need to double their dose from noon or afternoon may be in the prednisolone study period. As such, doubling their dose at noon would mean that they have taken a double dose of placebo. The only way to overcome this problem, is to ask the first dose to be doubled with a morning tablet, which will contain active ingredient regardless of the study period.

6.6 COVID-19 CONCURRENT INFECTION MANAGEMENT PLAN

If a participant on the study is diagnosed as asymptomatically positive for COVID-19, their management will continue unchanged on the study. This means that they will continue to take their IMP, unless the clinical circumstances change. Study visits will be delayed as necessary if there is a risk of transmission from an asymptomatic participant. Transmission risk will be assessed by a clinician on the study

Patients who are symptomatic and test positive for COVID-19, or who are symptomatic and are clinically assessed to have probable COVID-19 in the absence of a positive test, will be managed on a case-by-case basis according to clinical assessment with consideration of the current recommendations at the time. This may require the use of open-labelled prednisolone or hydrocortisone, meaning the temporary cessation of study IMP. Once the participant has clinically improved, the IMP will be restarted with a doubling regimen if deemed necessary. Remaining visits may be delayed accordingly, if the infection occurs close to a study Visit. Protocol deviation forms will be completed in these circumstances.

Emergency treatment is unchanged. Decisions on COVID-19 management will be made clinically on a case-by-case basis in accordance with the guidance above. The final decision in all cases will be made by the Chief Investigator.

7. PHARMACOVIGILANCE

7.1 DEFINITIONS

Adverse Event (AE): any untoward medical occurrence in a patient or clinical trial subject administered a medicinal product and which does not necessarily have a causal relationship with this treatment. An AE can therefore be any unfavourable and unintended sign (including an abnormal laboratory finding), symptom, or disease temporally associated with the use of an investigational medicinal product (IMP), whether or not considered related to the IMP.

Adverse Reaction (AR): all untoward and unintended responses to an IMP related to any dose administered. All AEs judged by either the reporting investigator or the sponsor as having reasonable causal relationship to a medicinal product qualify as adverse reactions. The expression reasonable causal relationship means to convey in general that there is evidence or argument to suggest a causal relationship.

Unexpected Adverse Reaction: an AR, the nature or severity of which is not consistent with the applicable product information (e.g. investigator's brochure for an unapproved investigational product or summary of product characteristics (SmPC) for an authorised product). When the outcome of the adverse reaction is not consistent with the applicable product information this adverse reaction should be considered as unexpected. Side effects documented in the SmPC which occur in a more severe form than anticipated are also considered to be unexpected.

Serious Adverse Event (SAE) or Serious Adverse Reaction: any untoward medical occurrence or effect that at any dose:

- **Results in death**.
- **Is life-threatening** – refers to an event in which the subject was at risk of death at the time of the event; it does not refer to an event which hypothetically might have caused death if it were more severe.

- **Requires hospitalisation, or prolongation of existing inpatients' hospitalisation.**
- **Results in persistent or significant disability or incapacity**.
- **Is a congenital anomaly or birth defect**.

Medical judgement should be exercised in deciding whether an AE/AR is serious in other situations. Important AE/ARs that are not immediately life-threatening or do not result in death or hospitalisation but may jeopardise the subject or may require intervention to prevent one of the other outcomes listed in the definition above, should also be considered serious.

Suspected Unexpected Serious Adverse Reaction (SUSAR): any suspected adverse reaction related to an IMP that is both unexpected and serious.

7.2 CAUSALITY

Most adverse events and adverse drug reactions that occur in this study, whether they are serious or not, will be expected treatment-related toxicities due to the drugs used in this study. The assignment of the causality should be made by the investigator responsible for the care of the participant using the definitions in the table below.

If any doubt about the causality exists, the local investigator should inform the study coordination centre who will notify the Chief Investigators. The pharmaceutical companies and/or other clinicians may be asked to advise in some cases.

In the case of discrepant views on causality between the investigator and others, all parties will discuss the case. In the event that no agreement is made, the MHRA will be informed of both points of view.

Relationship	Description
**Unrelated**	There is no evidence of any causal relationship
**Unlikely**	There is little evidence to suggest there is a causal relationship (e.g. the event did not occur within a reasonable time after administration of the trial medication). There is another reasonable explanation for the event (e.g. the participant's clinical condition, other concomitant treatment).
**Possible**	There is some evidence to suggest a causal relationship (e.g. because the event occurs within a reasonable time after administration of the trial medication). However, the influence of other factors may have contributed to the event (e.g. the participant's clinical condition, other concomitant treatments).
**Probable**	There is evidence to suggest a causal relationship and the influence of other factors is unlikely.
**Definitely**	There is clear evidence to suggest a causal relationship and other possible contributing factors can be ruled out.
**Not assessable**	There is insufficient or incomplete evidence to make a clinical judgement of the causal relationship.

7.3 REPORTING PROCEDURES

All adverse events should be reported. Depending on the nature of the event the reporting procedures below should be followed. Any questions concerning adverse event reporting should be directed to the study coordination centre in the first instance. A flowchart is given below to aid in the reporting procedures.

7.3.1 Non-serious AR/AEs

All such toxicities, whether expected or not, should be recorded in the toxicity section of the relevant case report form and sent to the study coordination centre within one month of the form being due.

7.3.2 Serious AR/AEs

All SAEs and SUSARs should be reported on the day that the local site is aware of the event. The SAE form asks for nature of event, date of onset, severity, corrective therapies given, outcome and causality (i.e. unrelated, unlikely, possible, probably, definitely). The responsible investigator should sign the causality of the event. Additional information should be sent within 5 days if the reaction has not resolved at the time of reporting.

SAEs
An SAE form should be completed and faxed or emailed to the study coordination centre and sponsor for all SAEs within 24 hours. However, relapse and death due to adrenal insufficiency, and hospitalisations for elective treatment of a pre-existing condition do not need reporting as SAEs.

SUSARs
In the case of suspected unexpected serious adverse reactions, the staff at the site should:

> Complete the SAE case report form & send it immediately (within 24 hours, preferably by fax), signed and dated to the study coordination centre and sponsor together with relevant treatment forms and anonymised copies of all relevant investigations.

Or

> Contact the study coordination centre by phone and then send the completed SAE form to the study coordination centre and sponsor within the following 24 hours as above.

The study coordination centre will notify the MHRA, REC and the Sponsor of all SUSARs occurring during the study according to the following timelines; fatal and life-threatening within 7 days of notification and non-life threatening within 15 days. All investigators will be informed of all SUSARs occurring throughout the study.

Local investigators should report any SUSARs and /or SAEs as required by their Local Research Ethics Committee and/or Research & Development Office.

7.4 REFERENCE SAFETY INFORMATION FOR HYDROCORTISONE

Undesirable effects:

Blood and Lymphatic System Disorders: Leucocytosis

Immune System Disorders: Hypersensitivity.

Endocrine Disorders: Increased or decreased motility and number of spermatozoa, menstrual irregularities, amenorrhoea, development of Cushingoid state, suppression of growth in children, secondary adrenocortical and pituitary unresponsiveness (particularly in times of stress, as in trauma, surgery, or illness), decreased carbohydrate tolerance, manifestations of latent diabetes mellitus, hyperglycaemia, increased requirements for insulin or oral hypoglycaemic agents in diabetes, hirsutism.

Metabolism & Nutrition Disorders: Sodium retention, fluid retention, potassium loss, hypokalaemic alkalosis, increased calcium excretion, negative nitrogen balance due to protein catabolism, weight gain, increased appetite.

Psychiatric Disorders: psychic disturbances, psychological dependence, insomnia. A wide range of psychiatric reactions including affective disorders (such as irritable, euphoric, depressed and labile mood, and suicidal thoughts), psychotic reactions (including mania, delusions, hallucinations and aggravation of schizophrenia), behavioural disturbances, irritability, anxiety, sleep disturbances, and cognitive dysfunction including confusion and amnesia have been reported. Reactions are common and may occur in both adults and children. In adults, the frequency of severe reactions has been estimated to be 5-6%. Psychological effects have been reported on withdrawal of corticosteroids; the frequency is unknown.

Nervous System Disorders: Convulsions, increased intracranial pressure with papilloedema (pseudotumour cerebri) usually after treatment, vertigo, headache, malaise.

Eye disorders: Posterior subcapsular cataracts, increased intra-ocular pressure, papilloedema, corneal or scleral thinning, exacerbation of ophthalmic viral disease, glaucoma, exophthalmos.

Gastro-intestinal Disorders: Peptic ulcer with possible perforation and haemorrhage, perforation of the small and large bowel particularly in patients with inflammatory bowel disease, pancreatitis, abdominal distension, ulcerative oesophagitis, dyspepsia, oesophageal candidiasis, nausea.

Skin and Subcutaneous Tissue Disorders: Impaired wound healing, thin fragile skin, petechiae, and ecchymoses, erythema, striae, telangiectasia, acne, increased sweating, may suppress reactions to skin tests, other cutaneous reactions such as allergic dermatitis, urticaria, angioneurotic oedema

Musculoskeletal, Connective Tissue & Bone Disorders: Muscle weakness, steroid myopathy, loss of muscle mass, osteoporosis (especially in postmenopausal females), vertebral compression fractures, aseptic necrosis of femoral and humeral heads, pathological fracture of long bones, tendon rupture.

Cardiac Disorders: Myocardial rupture following recent myocardial infarction, congestive heart failure in susceptible patients,

Vascular Disorders: thrombo-embolism, hypertension,

Respiratory, Thoracic & Mediastinal Disorders: Hiccups.

Other: Hypersensitivity, leucocytosis, weight gain, increased appetite, nausea, malaise.

7.5 REFERENCE SAFETY INFORMATION FOR PREDNISOLONE

Undesirable effects:

The following safety information applies to prednisolone when used as an anti-inflammatory. As prednisolone will be used at low doses in this study as steroid replacement therapy, these undesirable effects are not expected.

The incidence of predictable undesirable effects, including hypothalamic-pituitary-adrenal suppression correlates with the relative potency of the drug, dosage, timing of administration and the duration of treatment (see “other special warnings and precautions”). Undesirable effects may be minimised by using the lowest effective dose for the minimum period, and by administering the daily requirement as a single morning dose or whenever possible as a single morning dose on alternative days. Frequent patient review is required to appropriately titrate the dose against disease activity. Anti-inflammatory/immunosuppressive: Increased susceptibility and severity of infections with suppression of clinical symptoms and signs, opportunistic infections, recurrence of dormant tuberculosis.

Gastrointestinal: Abdominal distension, acute pancreatitis, dyspepsia, nausea, increased appetite, oesophageal candidiasis, oesophageal ulceration, peptic ulceration with perforation and haemorrhage, perforation of the small bowel, particularly in patients with inflammatory bowel disease.

Endocrine/metabolic: Cushingoid facies, growth suppression in infancy, childhood and adolescence, hirsutism, impaired carbohydrate tolerance with increased requirement for antidiabetic therapy, menstrual irregularity and amenorrhoea, negative protein and calcium balance, suppression of the hypothalamo-pituitary adrenal axis, and weight gain. Although the frequency is not known, there is a risk for Cushing Syndrome.

Fluid and electrolyte disturbance: Hypertension, nocturia, hypokalaemic alkalosis, potassium loss, sodium and water retention, risk of congestive heart failure in susceptible patients.

Musculoskeletal: Avascular osteonecrosis, osteoporosis, proximal myopathy, tendon rupture, vertebral and long bone fractures, muscle weakness, wasting and loss of muscle mass.

Dermatological: Acne, bruising, impaired healing, skin atrophy, striae, telangiectasia.

Neuropsychiatric: A wide range of psychiatric reactions including affective disorders (such as irritable, euphoric, depressed and labile mood, and suicidal thoughts), psychotic reactions (including mania, delusions, hallucinations, and aggravation of schizophrenia), marked euphoria leading to dependence; aggravation of epilepsy, behavioural disturbances, irritability, nervousness, anxiety, sleep disturbances, and cognitive dysfunction including confusion and amnesia have been reported. Reactions are common and may occur in both adults and children. In adults, the frequency of severe reactions has been estimated to be 5-6%. Psychological effects have been reported on withdrawal of corticosteroids; the frequency is unknown. Intracranial pressure with papilloedema in children (pseudotumour cerebri) usually after treatment withdrawal, psychological dependence.

Ophthalmic: Corneal or scleral thinning, scleral perforation, exacerbation of ophthalmic viral or fungal disease, glaucoma, increased intra-ocular pressure, papilloedema, posterior subcapsular cataracts, central serous chorioretinopathy (frequency not know known).

General: Hypersensitivity including anaphylaxis, leucocytosis, malaise, thromboembolism.

Withdrawal symptoms: Too rapid a reduction of corticosteroid dosage following prolonged treatment can lead to acute adrenal insufficiency, hypotension and death. A “withdrawal syndrome” may also occur including arthralgia, conjunctivitis, fever, loss of weight, myalgia, painful itchy skin nodules and rhinitis.

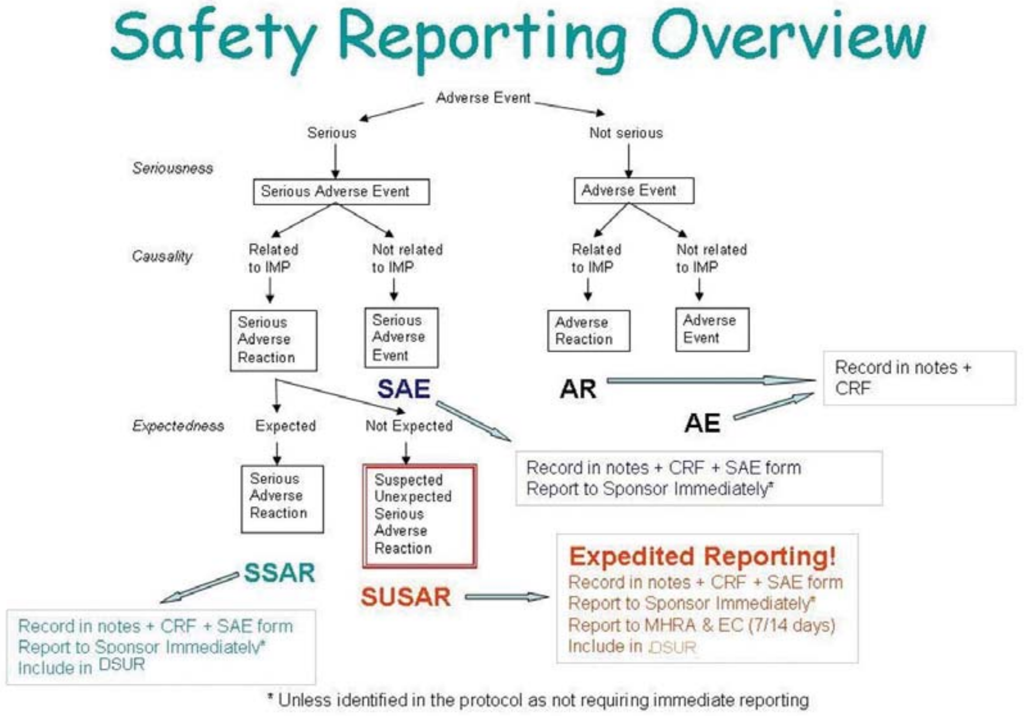

Contact details for reporting SAEs and SUSARs
RGIT.ctimp.team@imperial.ac.uk
Chief Investigator: k.meeran@imperial.ac.uk
Please send SAE forms to: Prof Karim Meeran, Section of Investigative Medicine, Imperial College London, 6th Floor Commonwealth Building, Du Cane Road, London W12 0NN
Tel: 020 3313 3052 (Mon to Fri 09.00 – 17.00) / 07555 717544
Email: steroids@imperial.ac.uk

8. ASSESSMENT AND FOLLOW-UP

Length of study: 9-month duration for each participant

Study visits:

There will be 3 planned study visits, and 4 scheduled telephone consultations during each study period (total of 6 study visits and 8 telephone consults over both periods). The study visits will

be completed on Day 1 and Day 30 in the first month, and the final study visit of each treatment arm on Day 120. Telephone consultations will be on Days 8 and 15 in the first month, followed by further monthly consults on Day 60 and Day 90. Please refer to Appendix 2 for a schematic of the time and events on each study visit. The time window for completing each study visit is ±3 days of the target date. Study visits which are completed outside of this window will require a protocol deviation form.

For each study visits, participants will be asked to attend the Clinical Research Facility at the Hammersmith Hospital at 0800h having fasted from 2200h the night before (permitting small sips of water only) and having taken their first steroid tablet of the day at least 2 hours prior. On arrival, observations including heart rate, blood pressure, weight and waist-hip circumferences will be recorded. Urine will be collected for measurement of additional bone markers. The urine will also be subjected to a pregnancy test in participants of child bearing potential. Routine safety blood tests, and immunological blood samples will be collected during each visit. Bone markers and glycaemic markers will also be measured. Participants will be asked to report any symptoms and to complete the SF-36, Addi-QoL and GNCQ questionnaires during each visit.

On the first day of each study period and during visit 1 (visit 4 in the second period), participants will take their usual baseline steroid replacement therapy. Participants will commence the blinded study medication from Day 2 until Day 120 (visit 3).

At the end of each study period, participants will be asked to return their remaining tablets, which will be used to gauge compliance.

8.1 LOSS TO FOLLOW-UP

In the instance that a participant is suspected to be lost to follow up, a member of the research team will initially attempt to make contact via telephone calls and email. After a reasonable attempt has been made to re-initiate contact, a letter will be sent to the participant by a study physician asking them to call, write or email the study team at their convenience within a week. If no response is received, a personalised letter will be sent to the participant's GP to inform them of the situation and in the interests of safety, to ask them to ensure that the patient has returned to their baseline steroid replacement therapy. In certain circumstances a phone call may be placed to the GP to advise them of the situation. The actions taken by the research team will be recorded in the participants NHS hospital notes.

The decision to withdraw a participant in these instances will be taken on a case-by-case basis by the Chief Investigator. If there are extenuating circumstances and the patient has continued their blinded medication, with the no compromise to the data collected and to the participant's safety, the participant may be permitted to continue on the study provided that they continue to consent. The Chief Investigator may opt to withdraw a patient if he feels that there is risk to the participant's safety or integrity of the data collected.

Data and samples collected on participants who are lost to follow up will be kept and used in the intention to treat analysis. The sample size calculation of 55 allows for a conservative 20% dropout rate. Participants lost to follow up will be replaced if required (where drop out exceeds 20%), to ensure the minimum power for analysis of this study is achieved.

8.2 TRIAL CLOSURE

The end of the study is defined as the completion of the last visit for the last participant. Once all queries are resolved the study will then be locked, unblinded and will enter a data analysis phase, before final closure of the study. This is anticipated to take 6 months.

The Chief Investigator will notify the sponsor the trial has ended. The REC and MHRA will be informed that the study has been concluded within 90 days (or 15 days in the case of a premature closure of the study).

All remaining IMPs will be disposed of by the ICHNT pharmacy or returned to Activase Pharmaceuticals Ltd, after accountability and inventory has been checked by the trial monitor. Authorisation should be sought from the sponsor or CI prior to disposal or return of the IMP.

When the study has been closed, the participants will be unblinded and the data analysis phase will begin. Code-break envelopes will be retrieved from all holders.

The trial will be halted prematurely if a particular statistically significant serious adverse event in more than three individuals upon review by the chief investigator or DMC is deemed to be related to either one of the study medications, and not the medical condition itself.

9. STATISTICS AND DATA ANALYSIS

Sample size

The intention is to recruit 55 participants, allowing for a 20% drop-out rate. With 44 completers analysed there is a minimum of 90% power to detect a 1.5 µg/L effect on osteocalcin with a two-sided test at the 5% level of significance, assuming an estimate of the SD at a single timepoint of 3.0 (14). This assumes cautiously that the correlation between the repeated measurements within patients across the two periods is 0.5, whereas a previous crossover trial(17) showed a correlation of 0.8.

Following a pilot study to elucidate a reference range for osteocalcin in healthy volunteers and patients with AI, it has been shown that individuals can demonstrate reductions of up to 2.4 µg/L in the short term after changes in their steroid replacement regimen. It is anticipated that that larger changes in osteocalcin will be seen when patients continue on their new regimens for a longer duration.

Methods of data collection and analysis

Data will be collected and recorded throughout the study on both paper case record files and a validated database (Oracle InForm). Following last patient last visit and all data entry final data checks will be conducted. Post the resolution of any final data queries database lock will occur for analysis.

The analysis will follow the intention-to-treat principle and include all available data from participants within the treatment sequences to which they were randomised, regardless of treatment subsequently received.

As this is a two-period two-treatment crossover trial, the treatment effect will be estimated using a linear mixed effect model with participant as a random effect and fixed effects for treatment

and period, type of AI (primary vs secondary). Model assumptions, including the normality of residuals and random effects distributions will be assessed. If any assumptions are poorly met, then an appropriate transformation of the outcome (e.g. log transformation) will be considered.

Every effort will be made to obtain all follow up data for all participants, including those that have stopped treatment. The analysis method employs maximum likelihood estimation and is thus efficient for handing missing outcome data under a missing at random (MAR) assumption. Where appropriate sensitivity analyses will be conducted to check that the conclusions are robust for, example, to the missing data assumption. Although a washout period is incorporated into the design of the study to minimise carry-over effects, we will assess whether there is any evidence of a carry-over effect by fitting a separate linear mixed model which further includes the treatment by period interaction.

The continuous secondary outcomes will be analysed using the same modelling approach as specified above for the primary outcome. Where there are repeated measures of an endpoint within each period, we will display the effects over time and choose further appropriate statistical methods to assess any differences in the patterns over time. We will report compliance to study drug using data for pills missed doses over the study period. Adverse events will be tabulated by treatment group and period.

All treatment effect estimates will be reported with 95% confidence intervals and a two-sided 5% level of significance will be used. A detailed statistical analysis plan will be drawn up and agreed by the TSC prior to any unblinded data extraction.

Methods of randomisation

Participants will be randomised in this study to either arm (A) or (B), using stratified blocked randomisation. Randomisation will be blocked for type of AI (primary vs secondary) to minimise heterogeneity.

Block size will vary randomly, ranging from 2 to 6.

Randomisation will be managed through the Oracle InForm data capture system as described above. Manual back-up lists will be held by an independent statistician at the ICTU for use if InForm is unavailable at any time. The InForm database will then be updated the reflect the new events at the earliest opportunity.

Data and all appropriate documentation will be stored for a minimum of 10 years after the completion of the study, including the follow-up period.

10. MONITORING

10.1 RISK ASSESSMENT

This study has undergone a risk assessment by the NIHR Imperial Clinical Research Facility, as part of the normal procedure to host this study. The trial is assessed to be high risk, by virtue of its double-blind crossover design and involvement of patients.

In addition to monitoring by the sponsor, the Trial Management Group (Professor Meeran, Professor Tan, and Dr Choudhury- subject to change) will convene on a weekly basis to review the day-to-day running of the study.

An independent data monitoring committee (DMC) will be chaired by Dr Benjamin Field. Dr Genevieve Wills of the MRC Clinical Trials Unit at UCL will serve as the independent statistician. Dr Narendra Reddy from University Hospitals of Leicester NHS Trust, will be an expert clinician member of the DMC. All clinical members have relevant expertise in the management of patients with AI. The role of this partially-blinded committee will be to review the safety data from the trial on a 6-monthly basis and to advise the Sponsor as to whether there are any ethical or safety reasons the study should not proceed, as per guidance from NIHR: (http://www.nets.nihr.ac.uk/__data/assets/pdf_file/0014/165110/NETSCC_TSC_SSC-Guidance_April-2016.pdf)

The trial steering committee (TSC) will be convened on a 6-monthly basis and will be chaired by an independent clinician, Dr Bernard Khoo. The committee will include Prof Maralyn Druce (The William Harvey Research Institute - Barts and The London, expert clinician), Prof Waljit Dhillo (Imperial) and two lay members, Mr Joel Russell-Winter and Mrs Gill Masters (from the charity, AMEND).

The DMC and TSC will be convened prior to initiation of the study or early in the recruitment phase, and will convene on a 6 monthly basis thereafter, unless either committee decide otherwise.

10.2 MONITORING AT STUDY COORDINATION CENTRE

Data will be collected by the study team and entered online into a trial specific Oracle InForm electronic data capture system (EDC). The database is built from requirements defined by the Chief Investigator and trial team. The InForm EDC system is built using Oracle Central Designer on an Oracle database. The InForm system will be built with data validation and edit checks as defined in the trial requirements; queries and alerts will be automatically generated as required. Monitoring will be performed electronically as defined in the trial monitoring plan. The InForm system is web based with role and site-based security applied

Captured data will also visually reviewed by members of the study at intermittent intervals as frequently as on a monthly basis. Any data entries that are suspect (unusual entries, missing entries, double entries) will be confirmed by consulting the source data and corrected.

11. REGULATORY ISSUES

11.1 CTA

This study has Clinical Trials Authorisation from the UK Competent Authority; MHRA. Reference: 19174/0407/001-0001

11.2 ETHICS APPROVAL

The Study Coordination Centre has obtained approval from the London-South East Research Ethics Committee (REC) and Health Regulator Authority (HRA). The study must also receive confirmation of capacity and capability from each participating NHS Trust before accepting

participants into the study or any research activity is carried out. The study will be conducted in accordance with the recommendations for physicians involved in research on human subjects adopted by the 18th World Medical Assembly, Helsinki 1964 and later revisions.

11.3 CONSENT

Consent to enter the study must be sought from each participant only after a full explanation has been given, an information leaflet offered, and time allowed for consideration. Signed participant consent should be obtained. The right of the participant to refuse to participate without giving reasons must be respected. After the participant has entered the trial the clinician remains free to give alternative treatment to that specified in the protocol at any stage if he/she feels it is in the participant's best interest, but the reasons for doing so should be recorded. In these cases, the participants remain within the study for the purposes of follow-up and data analysis. All participants are free to withdraw at any time from the protocol treatment without giving reasons and without prejudicing further treatment, but will be encouraged to provide the reason, which will be recorded, if they are happy to do so. Participants who choose to withdraw will be encouraged to continue to provide outcome data for the remaining trial visits for the current treatment period, even though they may no longer be taking the blinded medication.

11.4 CONFIDENTIALITY

Participants' identification data will be required for the registration process. The Study Coordination Centre will preserve the confidentiality of participants taking part in the study and is registered under the Data Protection Act.

11.5 INDEMNITY

Imperial College London holds negligent harm and non-negligent harm insurance policies which apply to this study.

11.6 SPONSOR

Imperial College London will act as the main Sponsor for this study. Delegated responsibilities will be assigned to the NHS trusts taking part in this study.

11.7 FUNDING

The NIHR and Imperial Healthcare Charity are funding this study.

Participants will be paid £30 for each study visit attended for their expenses.

11.8 AUDITS AND INSPECTIONS

The study may be subject to inspection and audit by Imperial College London under their remit as Sponsor, the Study Coordination Centre and other regulatory bodies to ensure adherence to GCP.

12. TRIAL MANAGEMENT

A Trial Management Group (TMG) will be appointed and will be responsible for overseeing the progress of the trial. The day-to-day management of the trial will be co-ordinated through the Section of Investigative Medicine Study Coordination Centre.

An independent data monitoring committee (DMC) will be chaired by Dr Benjamin Field. Dr Genevieve Wills of the MRC Clinical Trials Unit at UCL will serve as the independent statistician. Dr Narendra Reddy from University Hospitals of Leicester NHS Trust, will be an expert clinician member of the DMC.. All clinical members have relevant expertise in the management of patients with AI. The role of this partially-blinded committee will be to review the safety data from the trial on a 6-monthly basis and to advise the Sponsor as to whether there are any ethical or safety reasons the study should not proceed, as per guidance from NIHR: (http://www.nets.nihr.ac.uk/__data/assets/pdf_file/0014/165110/NETSCC_TSC_SSC-Guidance_April-2016.pdf)

The trial steering committee (TSC) will be convened on a 6-monthly basis and will be chaired by an independent clinician, Dr Bernard Khoo. Prof Maralyn Druce (The William Harvey Research Institute - Barts and The London, expert clinician), Prof Waljit Dhillo (Imperial) and two lay members, Mr Joel Russell-Winter and Mrs Gill Masters (from the charity, AMEND).

The DMC and TSC will be convened prior to initiation of the study or early in the recruitment phase, and will convene on a 6 monthly basis thereafter, unless either committee decide otherwise.

13. PUBLICATION POLICY

All publications and presentations relating to the study will be authorised by the Trial Management Group. The first publication of the trial results will be in the name of the Trial Management Group, if this does not conflict with the journal's policy. If there are named authors, these will include at least the trial's Chief Investigator. Authorship of parallel studies initiated outside of the Trial Management Group will be according to the individuals involved in the project but will acknowledge the contribution of the Trial Management Group and the Study Coordination Centre where permitted within the journal's policy.

14. REFERENCES

(1) Charmandari E, Nicolaides NC, Chrousos GP. Adrenal insufficiency. *Lancet (London, England).* 2014;383(9935): 2152-2167.

(2) Regal M, Paramo C, Sierra SM, Garcia-Mayor RV. Prevalence and incidence of hypopituitarism in an adult Caucasian population in northwestern Spain. *Clinical endocrinology.* 2001;55(6): 735-740.

(3) DUNLOP D. Eighty-Six Cases of Addison's Disease. *British medical journal.* 1963;2(5362): 887-891.

(4) Husebye ES, Allolio B, Arlt W, Badenhoop K, Bensing S, Betterle C, et al. Consensus statement on the diagnosis, treatment and follow-up of patients with primary adrenal insufficiency. *Journal of internal medicine.* 2014;275(2): 104-115.

(5) Bensing S, Brandt L, Tabaroj F, Sjoberg O, Nilsson B, Ekbom A, et al. Increased death risk and altered cancer incidence pattern in patients with isolated or combined autoimmune primary adrenocortical insufficiency. *Clinical endocrinology.* 2008;69(5): 697-704.

(6) Bergthorsdottir R, Leonsson-Zachrisson M, Oden A, Johannsson G. Premature mortality in patients with Addison's disease: a population-based study. *The Journal of clinical endocrinology and metabolism.* 2006;91(12): 4849-4853.

(7) Peacey SR, Guo CY, Robinson AM, Price A, Giles MA, Eastell R, et al. Glucocorticoid replacement therapy: are patients over treated and does it matter? *Clinical endocrinology.* 1997;46(3): 255-261.

(8) Erichsen MM, Lovas K, Fougner KJ, Svartberg J, Hauge ER, Bollerslev J, et al. Normal overall mortality rate in Addison's disease, but young patients are at risk of premature death. *European journal of endocrinology / European Federation of Endocrine Societies.* 2009;160(2): 233-237.

(9) Shenfield GM, Paterson JW, Costello JF, Ijaduola O. The effect of prednisone treatment on the half-life of intravenous hydrocortisone. *British journal of clinical pharmacology.* 1974;1(3): 237-240.

(10) Bornstein SR, Allolio B, Arlt W, Barthel A, Don-Wauchope A, Hammer GD, et al. Diagnosis and Treatment of Primary Adrenal Insufficiency: An Endocrine Society Clinical Practice Guideline. *The Journal of clinical endocrinology and metabolism.* 2016;101(2): 364-389.

(11) Jodar E, Valdepenas MP, Martinez G, Jara A, Hawkins F. Long-term follow-up of bone mineral density in Addison's disease. *Clinical endocrinology.* 2003;58(5): 617-620.

(12) Dharmshaktu P, Aggarwal A, Dutta D, Kulshreshtha B. Bilateral femoral head avascular necrosis with a very low dose of oral corticosteroid used for panhypopituitarism. *BMJ case reports.* 2016;201610.1136/bcr-2015-212803.

(13) Lovas K, Gjesdal CG, Christensen M, Wolff AB, Almas B, Svartberg J, et al. Glucocorticoid replacement therapy and pharmacogenetics in Addison's disease: effects on bone. *European journal of endocrinology.* 2009;160(6): 993-1002.

(14) Koetz KR, Ventz M, Diederich S, Quinkler M. Bone mineral density is not significantly reduced in adult patients on low-dose glucocorticoid replacement therapy. *The Journal of clinical endocrinology and metabolism.* 2012;97(1): 85-92.

(15) Caldato MC, Fernandes VT, Kater CE. One-year clinical evaluation of single morning dose prednisolone therapy for 21-hydroxylase deficiency. *Arquivos Brasileiros de Endocrinologia e Metabologia.* 2004;48(5): 705-712.

(16) Machenahalli P, Choudhury S, Meeran K. Prednisolone 3 mg once daily should be the glucocorticoid replacement for hypopituitarism. *Endocrine Abstracts.* 2016;44(EP6): .

(17) Hunt PJ, Gurnell EM, Huppert FA, Richards C, Prevost AT, Wass JA, et al. Improvement in mood and fatigue after dehydroepiandrosterone replacement in Addison's

disease in a randomized, double blind trial. *The Journal of clinical endocrinology and metabolism.* 2000;85(12): 4650-4656.

(18) Benson S, Neumann P, Unger N, Schedlowski M, Mann K, Elsenbruch S, et al. Effects of standard glucocorticoid replacement therapies on subjective well-being: a randomized, double-blind, crossover study in patients with secondary adrenal insufficiency. *European journal of endocrinology.* 2012;167(5): 679-685.

(19) Oksnes M, Bensing S, Hulting AL, Kampe O, Hackemann A, Meyer G, et al. Quality of life in European patients with Addison's disease: validity of the disease-specific questionnaire AddiQoL. *The Journal of clinical endocrinology and metabolism.* 2012;97(2): 568-576.

Appendix 1: Schematic of pre-randomisation events Volunteers will be counselled about the study and receive a medical review during the screening visit. Blood samples, NTX quantification and questionnaires will only be completed if a volunteers is still eligible for the study following the medical review. This is to prevent unnecessarily performing venepuncture or wasting the volunteer's time.

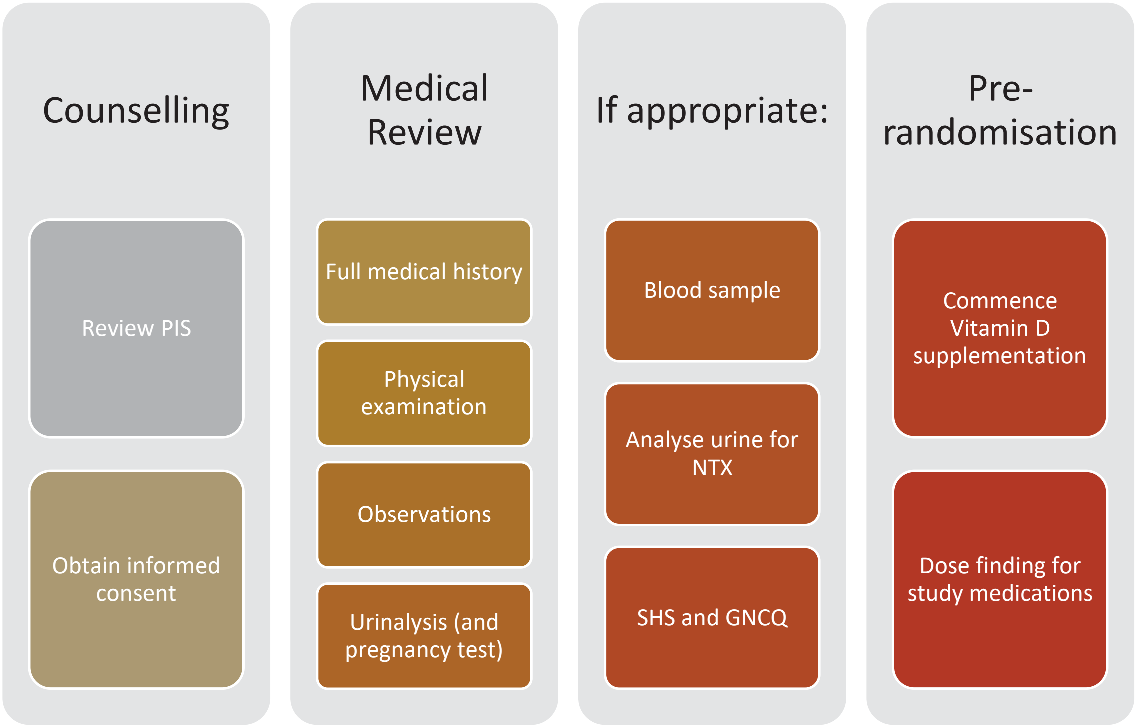

SHS:Subjective Health Status (including the short form health survey (SF-36) and AddiQoL); **GNCQ**: German National Cohort Questionnaire; **Blood profile 1**:fasting renal, bone and lipid profiles, bicarbonate, full blood count (FBC), glucose, insulin, fructosamine, HbA1c, creatine kinase(**CK**), Adrenocorticotropic Hormone (ACTH), cortisol binding globulin (CBG), parathyroid hormone (PTH), vitamin D, bone-specific alkaline phosphatase (**BALP**), osteocalcin (OC), procollagentype1 N-terminal propeptide(**P1NP**); hs-CRP, hs-Troponin I, BNP, assessment of soluble immunological antigens and assessment of white cell populations.

Appendix 2: schematic of study periods and visits Participants will attend visit 1 on their baseline therapy in both periods. They will be issued with steroid/placebo tablets that are identical in appearance. On Day 2, they will be instructed to take the tablets at the stated set times for a period of 4 months in the first period. This is followed by a 2-week minimum washout period on their baseline steroid replacement therapy, before commencing the second period. Patients will be on baseline therapy for Visit 1 of the second period prior to starting the blinded therapy on Day 2 for 4 months. Patients will randomised to undertake either arm (A) or (B). Arm (A) will involve administration of prednisolone in the first period and hydrocortisone in the second. Arm (B) will involve hydrocortisone in the first period and prednisolone in the second period. The example schematic below is for a participant on arm (A).

Study Period	Drug- Arm (A)	On Waking	Lunchtime (1200h)	Late afternoon (1700h)
1	Prednisolone	2-5mg	Placebo	Placebo
2	Hydrocortisone	10mg	5mg	5mg

First Study Period (e.g. Arm (A)- Prednisolone/Placebo/Placebo)									
**Time**	**Study Visit 1 Month 1 Day 1**	**Month 1 Day 2**	**Telephone Consult 1 Month 1 Day 8**	**Telephone Consult 2 Month 1 Day 15**	**Study Visit 2 Month 1 Day 30**	**Telephone Consult 3 Month 2 Day 60**	**Telephone Consult 4 Month 3 Day 90**	**Study Visit 3 Month 4 Day 120**	**2-week minimum washout period on baseline therapy**
06:00	Patient takes 1st tablet of the day at home (Baseline therapy)	Patient starts blinded medication at home			Patient takes 1st tablet of the day at home (Blinded treatment)			Patient takes 1st tablet of the day at home (Blinded treatment)	
08:00	Record weight, BP, heart rate, waist and hip circumference				Record weight, BP, heart rate, waist and hip circumference			Record weight, BP, heart rate, waist and hip circumference	
08:20	Obtain urine sample (NTX & pregnancy test)				Obtain urine sample (NTX & pregnancy test)			Obtain urine sample (NTX & pregnancy test)	
08:40	Blood profile 1				Blood profile 1			Blood profile 1	
08:55	AE reporting		AE reporting	AE reporting	AE reporting	AE reporting	AE reporting	AE reporting	
09:15	SHS surveys/GNCQ				SHS surveys/GNCQ			SHS surveys/GNCQ	

Second Study Period (e.g. Arm (A)- Hydrocortisone 10mg / 5mg / 5mg)									
**Time**	**Study Visit 1 (4) Month 1 Day 1**	**Month 1 Day 2**	**Telephone Consult 1 (5) Month 1 Day 8**	**Telephone Consult 2 (6) Month 1 Day 15**	**Study Visit 2 (5) Month 1 Day 30**	**Telephone Consult 3 (7) Month 2 Day 60**	**Telephone Consult 4 (8) Month 3 Day 90**	**Study Visit 3 (6) Month 4 Day 120**	**Study complete (Patient returns to baseline therapy)**
06:00	Patient takes 1st tablet of the day at home (Baseline therapy)	Patient starts blinded medication at home			Patient takes 1st tablet of the day at home (Blinded treatment)			Patient takes 1st tablet of the day at home (Blinded treatment)	
08:00	Record weight, BP, heart rate, waist and hip circumference				Record weight, BP, heart rate, waist and hip circumference			Record weight, BP, heart rate, waist and hip circumference	
08:20	Obtain urine sample (NTX & pregnancy test)				Obtain urine sample (NTX & pregnancy test)			Obtain urine sample (NTX & pregnancy test)	
08:40	Blood profile 1				Blood profile 1			Blood profile 1	
08:55	AE reporting		AE reporting	AE reporting	AE reporting	AE reporting	AE reporting	AE reporting	
09:15	SHS surveys/GNCQ				SHS surveys/GNCQ			SHS surveys/GNCQ	

AE: Adverse Event; **SHS**:Subjective Health Status (including the short form health survey (SF-36) and AddiQoL); **GNCQ**: German National Cohort Questionnaire; **Blood profile 1**:fasting renal, bone and lipid profiles, bicarbonate, full blood count (FBC), glucose, insulin, fructosamine, HbA1c, creatine kinase(**CK**), Adrenocorticotropic Hormone (ACTH), cortisol binding globulin (CBG), parathyroid hormone (PTH), vitamin D, bone-specific alkaline phosphatase (**BALP**), osteocalcin (OC), procollagentype1 N-terminal propeptide(**P1NP**); hs-CRP, hs-Troponin I, BNP, assessment of soluble immunological antigens and assessment of white cell populations.

Statistical Analysis Plan (SAP)

Safety and Efficacy of Prednisolone in Adrenal Insufficiency Disease (PRED-AID Study)

Version 1.0

1. APPROVAL SIGNATURES

Study Investigators:

Chief Investigator: Prof Karim Meeran, Section of Investigative Medicine, Imperial College London

Signature: Karim Meeran Date: 17/7/19

Co-investigator: Dr Sirazum Choudhury, Section of Investigative Medicine, Imperial College London

Signature: [illegible] Date: 17/7/19

Co-investigator: Prof Tricia Tan, Section of Investigative Medicine, Imperial College London

Signature: [illegible] Date: 17/7/19

Statistician: Dr Suzie Cro, Imperial Clinical Trials Unit

Signature: [illegible] Date: 17/7/19

SAP Working Group:

Dr Sirazum Choudhury

Dr Suzie Cro

Document version history log:

Version	Date	Author	Summary of changes made
1.0	09/04/2019	Sirazum Choudhury	First version

PRED-AID Study Protocol Version 1.0

2. CONTENTS

Table of Contents

1. **Approval Signatures**

2. **Contents**

3. **Abbreviations**

4. **Introduction/Study summary**

5. **Study Objectives**
5.1 Primary Objective

6. **Design**
6.1 Study Design
6.2 Treatment Arms
6.3 Study Population
6.4 Inclusion Criteria
6.5 Exclusion Criteria
6.6 Blinding
6.7 Sample Size
6.8 Schedule of Time and Events
6.9 Randomisation

7. **Populations of Analysis Sets**
7.1 Intent-to-Treat/randomised Population
7.2 Per protocol Population

8. **Variables of Analysis**
8.1 Primary Efficacy Variable
8.2 Secondary Efficacy Variables
8.3 Safety Variables
8.4 Demographic Variables

9. **Statistical Methodology**
9.1 General Methodology
9.2 Patient Flow (CONSORT diagram)
9.3 Baseline Demographics
9.4 Descriptive Analysis

9.5 Primary Efficacy Analysis
9.6 Secondary Efficacy Analysis
9.7 Safety Analysis
9.8 Sensitivity Analysis
9.9 Supplementary Analysis
9.10 Subgroup Analysis

10. References

11. Appendices
11.1 Appendix 1

3. ABBREVIATIONS

**ACTH**	Adrenocorticorticotropic hormone- pituitary hormone which modulates cortisol
**Addi-QoL**	Addison's disease specific Quality of Life Questionnaire- a subjective health questionnaire validated in adrenal insufficiency
**AE**	Adverse event
**AI**	Adrenal insufficiency- Condition in which individuals are unable to synthesise steroid hormones such as cortisol, a stress hormone. Without treatment, this condition is fatal.
**AR**	Adverse Reaction
**BALP**	Bone specific Alkaline Phosphatase- a bone formation marker
**BMI**	Body Mass Index
**BNP**	Brain Natriuretic Peptide a cardiac marker
**CBG**	Cortisol Binding Globulin- a protein which binds cortisol in the blood
**CI**	Chief Investigator
**CK**	Creatine Kinase- a muscle enzyme used to indicate muscle damage
**FBC**	Full Blood Count
**GNCQ**	German National Cohort Questionnaire- a measurement tool for infection rates
**HbA1c**	Haemoglobin A1c- a diabetic marker
**HOMA-IR**	Homeostatic Model Assessment for Insulin Resistance- an indicator of insulin sensitivity
**hs-CRP**	High sensitivity C-reactive Protein a cardiovascular risk marker
**ICHNT**	Imperial College Healthcare NHS Trust- Incorporates St Mary's Hospital, Hammersmith Hospital and Charing Cross Hospital
**ICTU**	Imperial Clinical Trials Unit
**IMP**	Investigational Medicinal Product
**ITT**	Intention To Treat
**IQR**	Interquartile Range
**LFT**	Liver Function Tests
**MAR**	Missing At Random
**NIHR**	National Institute of Health Research
**NTX**	N-Telopeptide Crosslinks- a bone reabsorption marker

**OC**	Osteocalcin- a bone formation marker
**P1NP**	Type 1 Pro-collagen N-terminal peptide- a bone formation marker
**PP**	Per Protocol
**PTH**	Parathyroid Hormone
**SAE**	Serious Adverse Event
**SAR**	Serious Adverse Reaction
**SD**	Standard Deviation
**SF-36**	Short Form Health Survey-36- a subjective health questionnaire
**SS**	Safety Set- a population of all participants who have received at least one dose of the assigned intervention, defined for the purpose of describing AEs
**SUSAR**	Suspected Unexpected Adverse Reaction
**TFT**	Thyroid Function tests

4. INTRODUCTION/STUDY SUMMARY

Steroid replacement therapy is vital for the health of patients with adrenal insufficiency (AI), who are unable to produce the natural stress hormone, cortisol. The objectives of steroid replacement therapy are to replace the body's physiological requirements for cortisol without over-replacement and consequent Cushing's syndrome. Equally, under-replacement presents the risk of patients experiencing potentially fatal Addisonian crises(1). Appropriately replacing a patient's steroid requirement is a significant challenge(2).

Hydrocortisone is used in the majority of patients with AI in the UK(3). However, hydrocortisone has a short duration of action, necessitating dosing 3 times a day(4). Low-dose prednisolone is an alternative to hydrocortisone which needs only once-daily. There have been no studies directly comparing low-dose prednisolone to hydrocortisone treatment.

Using a two-arm, two-period, double-blind randomised crossover study design, this study aims to compare the effects of two routine treatments of AI, low dose prednisolone and standard regimens of hydrocortisone. The primary outcome will be bone health. Secondary outcomes include: Cardiovascular risk, Glycaemic contron, Infection rates and severity, Immunology profiles, Adverse events and Adverse Reactions, Wellbeing and Compliance

5. STUDY OBJECTIVES

5.1 PRIMARY OBJECTIVE

To evaluate and compare the safety and efficacy of low dose prednisolone and hydrocortisone treatments for the management of AI.

6. DESIGN

6.1 STUDY DESIGN

This study will be a phase 3, two-period, randomised cross-over, double-blind study with two treatment arms:

A) Participants given once daily low dose prednisolone (at 2-5 mg, determined by the serum level of prednisolone at 8 hours after a test dose) with placebo tablets at lunch and in the afternoon for four months (first period); followed by hydrocortisone three times daily (at their stable therapeutic regimen or the dose identified post-screening) for four months in the second period

B) Participants given hydrocortisone three times daily (at their stable therapeutic regimen or the dose identified post-screening) for four months (first period); followed by given once daily low dose prednisolone (at 2-5 mg, determined by the serum level of prednisolone at 8 hours after a test dose) with placebo tablets at lunch and in the afternoon for four months in the second period

Participants will be randomly assigned to begin with either treatment arm. Each treatment period will be continued for 4 consecutive months. Participants will then undergo a washout period during which they will be returned to their baseline treatment for a period of 2 weeks, before starting on the alternate treatment arm. The aim is to assess the effect of each treatment arm on bone health, surrogate markers of cardiovascular risk, glycaemic control, infection rates, immunology profiles, patient wellbeing and compliance. A schematic of the Study Visits, timeline and study events to be undertaken can be found in Appendix 1.

6.2 TREATMENT ARMS

At randomisation, participants will be allocated to one of two treatment arms, to receive either prednisolone therapy first, or hydrocortisone therapy first:

A) Prednisolone first: participants will receive four months of prednisolone and placebo tablets in the first Study Period and hydrocortisone in the second, four-month Study Period.

B) Hydrocortisone first: participants will receive four months of hydrocortisone in the first Study Period and prednisolone with placebo tablets in the second, four-month Study Period.

The dose of the participant's usual regimen will be unchanged from their pre-study dose. The dose of the alternative medication to be used will be elucidated prior to each participant's randomisation on the study.

6.3 STUDY POPULATION

The study population is patients with either primary (affecting the adrenal glands) or secondary (affecting the pituitary gland) AI, who require cortisol replacement treatment using prednisolone, hydrocortisone or other glucocorticoid.

6.4 INCLUSION CRITERIA

- Aged 18 – 70 years
- Male or female
- Diagnosed with AI for over 6 months according to standard diagnostic criteria
- Established on stable HC replacement or prednisolone replacement, dose not altered for at least 3 months
- Established on a stable dose of Fludrocortisone, if taking, dose not altered for at least 3 months
- Participants taking other hormone replacements (e.g. levothyroxine, testosterone or growth hormone in secondary adrenal insufficiency) are accepted providing that their replacement doses have not altered for at least 3 months
- Participants who are otherwise healthy enough to participate, as determined by pre-study medical history and physical examination.

- Participants who are able and willing to give written informed consent to participate in the study.

6.5 EXCLUSION CRITERIA

- Participants with a diagnosis of Type 1 or Type 2 diabetes mellitus.
- Unable to give informed consent.
- Taking supplements or herbal medications that the participant is unwilling or unable to stop prior to and during the study period e.g. St John's Wort (may decrease prednisolone levels), Cat's claw, Echinacea (immunomodulatory properties).
- Currently taking medications that alter CYP3A4 metabolism of glucocorticoids that the participant is unwilling or unable to stop prior to and during the study period e.g. phenytoin, phenobarbital, rifampicin, rifabutin, carbamazepine, primidone, aminoglutethimide, itraconazole, ketoconazole, ciclosporin or ritonavir.
- Pregnancy, taking the combined oral contraceptive pill, or oral oestrogen replacement therapy due to the effects on cortisol binding globulin levels and determination of prednisolone levels. Transdermal oestrogen replacement is permitted.
- Diagnosis of congenital adrenal hyperplasia, untreated

6.6 BLINDING

This is a double-blind study. To achieve sufficient blinding, patients will receive an individual tablet specially made for this study to be taken at their usual dosing times. Whilst taking hydrocortisone, patients will receive a hydrocortisone tablet in the morning, at noon, and in the afternoon, at their appropriate respective doses. When on prednisolone, patients will receive a single prednisolone tablet in the morning, at the appropriate dose. They will then receive placebo tablets at noon and in the afternoon.

In the case of a medical emergency or in the event of a serious medical condition, when knowledge of treatment allocation is essential for the clinical management or welfare of the participant, an investigator or other physician managing the subject may decide to unblind that subject's treatment code.

6.7 SAMPLE SIZE

The intention is to recruit 55 participants, allowing for a 20% drop-out rate. With 44 completers analysed there is a minimum of 90% power to detect a 1.5 µg/L effect on osteocalcin with a two-sided test at the 5% level of significance, assuming an estimate of the SD at a single timepoint of 3.0 (5). This assumes cautiously that the correlation between the repeated measurements within patients across the two periods is 0.5, whereas a previous crossover trial(6) showed a correlation of 0.8.

Following a pilot study to elucidate a reference range for osteocalcin in healthy volunteers and patients with AI, it has been shown that individuals can demonstrate reductions of up to 2.4 µg/L in the short

term after changes in their steroid replacement regimen. It is anticipated that that larger changes in osteocalcin will be seen when patients continue on their new regimens for a longer duration.

6.8 SCHEDULE OF TIME AND EVENTS

Pre-randomisation events are discussed in detail in the study protocol. The study will last 9 months for each participant. This will involve taking the first randomised treatment for 4 months in Study Period 1, followed by a 2-week washout period where patients return to their baseline therapy, and finally Study Period 2. Study Period 2 will also be a 4-month period in which the patient will take the alternative treatment to that used in Period 1.

There will be 3 planned study visits, and 4 scheduled telephone consultations during each Study Period (total of 6 study visits and 8 telephone consults over both periods). The study visits will be completed on Day 1 and Day 30 in the first month, and the final study visit of each treatment arm on Day 120. Telephone consultations will be on Days 8 and 15 in the first month, followed by further monthly consults on Day 60 and Day 90. Please refer to Appendix 1 for a schematic of the time and events on each study visit.

For each study visits, participants will be asked to attend the Clinical Research Facility at the Hammersmith Hospital at 0800h having fasted from 2200h the night before (permitting small sips of water only) and having taken their first steroid tablet of the day at least 2 hours prior. On arrival, observations including heart rate, blood pressure, weight and waist-hip circumferences will be recorded. Urine will be collected for measurement of additional bone markers. The urine will also be subjected to a pregnancy test in participants of child bearing potential. Routine safety blood tests, and immunological blood samples will be collected during each visit. Bone markers and glycaemic markers will also be measured. Participants will be asked to report any symptoms and to complete the SF-36, Addi-QoL and GNCQ questionnaires during each visit.

On the first day of each study period and during visit 1 (visit 4 in the second period), participants will take their usual baseline steroid replacement therapy. Participants will commence the blinded study medication from Day 2 until Day 120 (visit 3).

At the end of each study period, participants will be asked to return their remaining tablets, which will be used to gauge compliance.

6.9 Randomisation

Randomisation will be managed through the Oracle InForm data capture system. This is a validated GCP-compliant data capture and management system. The allocation to study arm will be based on the original randomisation list populated and held by an independent statistician at the ICTU, which will be available for reference and back-up if the InForm database is unavailable.

Participant demographic data will be uploaded to the InForm system at enrolment, when they will be assigned a Study ID. Participants will be randomised to a study arm, receiving a Study Drug ID based on their blocking factor. The Imperial pharmacy will be made aware of the allocation and will dispense the appropriate blinded study medication and specified placebo at the patient's individual dose, for the relevant study period.

Participants will be randomised in this study to either: (A) having prednisolone first or; (B) having hydrocortisone, using stratified blocked randomisation. Randomisation will be blocked for type of AI (primary vs secondary) to minimise heterogeneity. Given stratification for type of AI, all analyses will be adjusted for type of AI. Block size will vary randomly, ranging from 2 to 6.

7. POPULATIONS OF ANALYSIS SETS

7.1 INTENTION-TO-TREAT/RANDOMISED POPULATION

The main analysis will follow the intention-to-treat principle (ITT). All available primary and secondary outcomes from eligible participants who have been randomised will be analysed within the treatment group to which they were randomised for the associated period, regardless of treatment subsequently received.

7.2 PER PROTOCOL POPULATION

A secondary analysis will be performed on the per protocol (PP) population. All primary and secondary outcomes from participants who have completed all study visits successfully without unblinding or significant protocol deviations/violations (including non-compliance with medication) will be analysed.

The significance of protocol deviations/violations will be assessed by the trial management group, and the final decision as to whether a participant should be excluded from the per protocol analysis will lie with the Chief Investigator (CI).

8. VARIABLES OF ANALYSIS

8.1 PRIMARY EFFICACY VARIABLES

To evaluate the effects of two routine treatments of AI, low dose prednisolone and standard regimens of hydrocortisone on:

- Bone health
    - assessed by measurement of change in total osteocalcin and undercarboxylated osteocalcin, bone formation markers, between Day 1 (pre-blinded treatment) and Day 120 (4 months post blinded treatment).

8.2 SECONDARY EFFICACY VARIABLES

To compare the effects of two routine treatments of AI, low dose prednisolone and standard regimens of hydrocortisone between Day 1 (pre-blinded treatment) and Day 120 (4 months post blinded treatment):

- Other markers of bone health
    - assessed by measurement of change in additional bone markers and bone profile including procollagen type-1 N-terminal propeptide (P1NP), bone specific alkaline phosphatase (BALP), corrected calcium, parathyroid hormone (PTH), vitamin D and urinary N-terminal telopeptide (NTX).

- Surrogate markers and risk factors for cardiovascular disease
  - including anthropometric markers such as: blood pressure, heart rate, BMI (height on first occasion), weight and waist-hip circumference ratio.
  - cardiovascular risk assessed by measurement of high-sensitivity CRP (hs-CRP), high-sensitivity troponin I, lipid profile and BNP.

- Glycaemic control
  - assessed by HbA1c, fructosamine, fasting glucose levels and insulin resistance represented by Homeostatic Model Assessment for Insulin Resistance (HOMA-IR)

- Infection rates and severity
  - assessed by completion of the German National Cohort Questionnaire (GNCQ)

- Immunology profiles
  - Assessed by measurement and assessment of soluble immunological analytes such as CD16 and isolated white cell populations including total white cells, neutrophils, lymphocytes, eosinophils, basophils, natural killer cells, and monocytes (including subtypes).

- Safety
  - assessed by reporting of symptoms of steroid deficiency and myopathy and review of routine monitoring blood tests including full blood count (FBC), renal profile, liver function tests (LFTs), creatine kinase (CK), Adrenocorticotropic hormone (ACTH) cortisol binding globulin (CBG) and bicarbonate.

- Wellbeing
  - assessed by subjective health questionnaires including the SF-36 (7) and Addi-QoL (8)

- Compliance to regimen
  - assessed by collecting the remaining unused tablets at the end of each treatment arm

8.3 SAFETY VARIABLES

The safety variables measured in this study are included as part of the secondary efficacy variable and are reproduced below:

- Safety
  - assessed by reporting of symptoms of steroid deficiency and myopathy and review of routine monitoring blood tests including full blood count (FBC), renal profile, liver function tests (LFTs), creatine kinase (CK), Adrenocorticotropic hormone (ACTH) cortisol binding globulin (CBG) and bicarbonate.

In addition, the number of adverse events (AE), serious adverse events (SAE), and SUSARs (suspected unexpected serious adverse reactions) will be recorded for each Study Period.

8.4 DEMOGRAPHIC VARIABLES

The demographic variables to be considered in the analysis will at least include:

- Age
- Gender
- Weight
- BMI
- Cumulative daily dose of prednisolone required
- Cumulative daily dose of hydrocortisone required
- Type of AI (primary versus secondary)
- Number of anti-hypertensive agents (0, 1, 2, ≥3)
- Use of a lipid agent
- Use of a bone sparing agent

9. STATISTICAL METHODOLOGY

9.1 GENERAL METHODOLOGY

Data will be collected and recorded throughout the study on both paper case record files and a validated database (Oracle InForm). Following last patient last visit and all data entry final data checks will be conducted. Post the resolution of any final data queries database lock will occur for analysis.

The main analysis will be conducted following the intention-to-treat principle and include all randomised patients, according to their randomised assignment for each period, irrespective of treatment actually received. A supplementary secondary per-protocol analysis will be performed for the primary outcome.

Since randomisation is stratified by type of AI (primary vs secondary) analyses of outcomes will involve adjustment for this factor, unless otherwise indicated. For continuous outcomes, baseline values of the outcome will also be adjusted for to increase power(9). For treatment period 1 the baseline outcome will be the outcome measured at the start of period 1. For period 2 the baseline should be from the assessments measure after the completion of period 1 and washout. This baseline definition will be applied to all analyses. Change from baseline will be calculated as post-baseline value – baseline value for the associated period.

Missing baseline covariate data are not anticipated since covariates must be recorded to allocate treatment. However, if there are missing values for baseline measure of a clinical outcome, they will be replaced by the mean of the observed baseline values for all participants in all treatment arms (mean imputation). This technique improves the statistical efficiency in the estimation of treatment effect and is justifiable since randomisation ensures that baselines are independent of treatment group(10).

Every effort will be made to obtain all follow up data for all participants, including those that have stopped treatment. All available data will be included within the analysis. The primary analysis model to be employed uses maximum likelihood estimation and is consequently efficient for handing missing outcome data under a MAR assumption. Where appropriate sensitivity analyses will be conducted to check that the conclusions are robust for, example, to the missing data assumption.

Although a washout period is incorporated into the design of the study to minimise carry-over effects, we will assess whether there is any evidence of a carry-over effect by fitting a separate linear mixed model which further includes the treatment by period interaction.

The continuous secondary outcomes will be analysed using the same modelling approach as specified for the primary outcome.

All treatment effect estimates will be reported with 95% confidence intervals. A two-sided 5% level of significance will be used to assess the primary outcome. Statistical analysis will be conducted using SPSS or Stata.

9.2 PATIENT FLOW (CONSORT DIAGRAM)

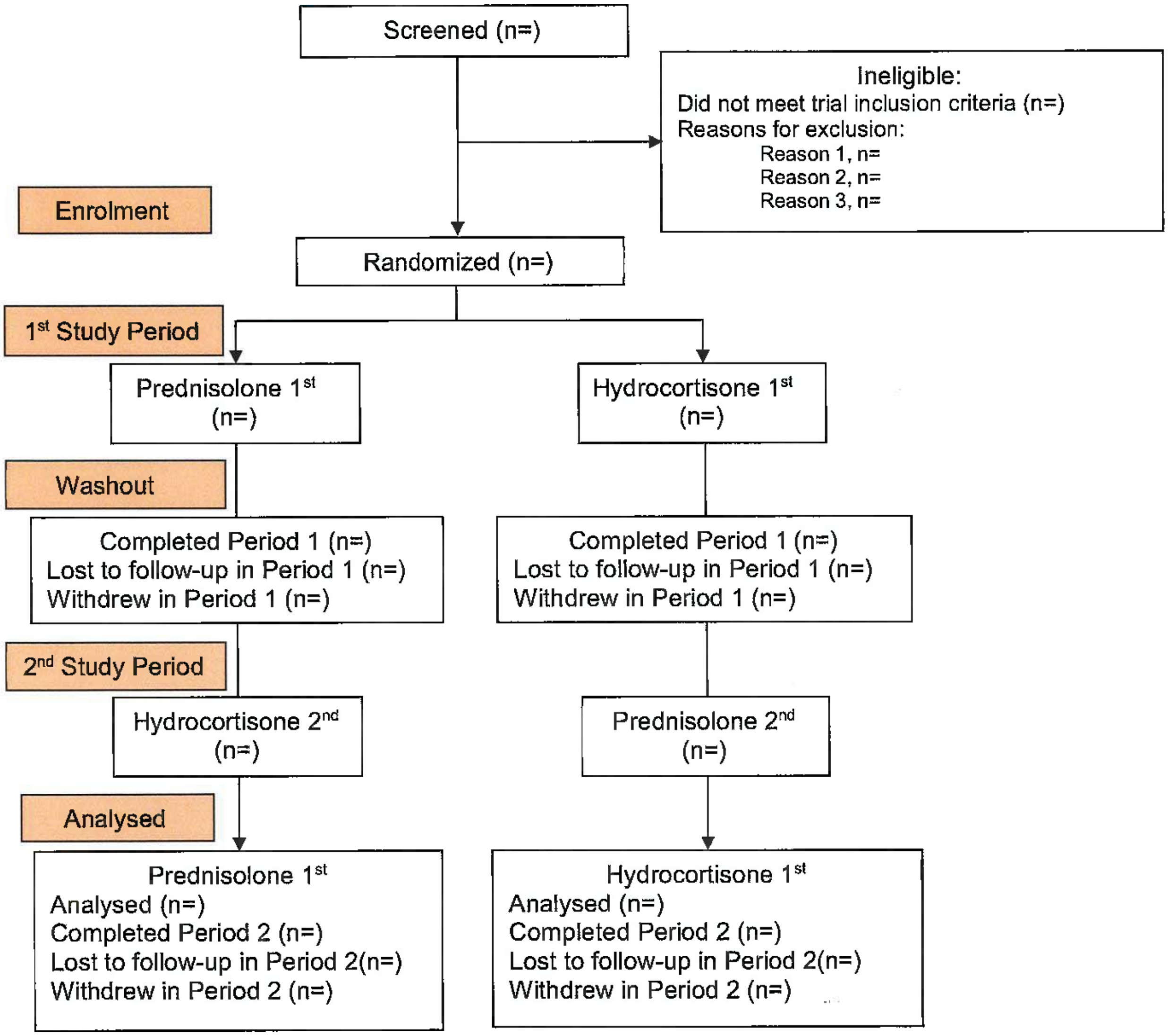

9.3 BASELINE DEMOGRAPHICS

Baseline characteristics will be reported according to treatment arm. Normally distributed continuous variables will be conveyed as mean (Standard Deviation (SD)), and median (Interquartile Range (IQR)) if non-normal. Categorical data will be reported using frequencies and proportions (as a %). The summaries will be based on observations only and the number of missing observations will be reported. No formal statistical tests will be performed since any differences between treatment arms at baseline will be the result of chance rather than bias, due to randomisation.

Concomitant medication will also be tabulated by treatment arm according to the general classification of the medication, as described in the British National Formulary (BNF). The number of participants in each arm using each medication will be reported.

9.4 DESCRIPTIVE ANALYSIS

Descriptive statistics will be presented for the primary and secondary outcome measures by treatment arm, period and time point.

9.5 PRIMARY EFFICACY ANALYSIS

As this is a two-period two-treatment crossover trial, the treatment effect will be estimated using a multi-level repeated measures linear mixed effect model. The change from baseline osteocalcin will be the dependent variable, at 30 days and 120 days. The model will include participant as a random effect and fixed effects for treatment and period, type of AI (primary vs secondary), baseline osteocalcin, use of a bone sparing agent, and time point (Day 30 and Day 120). An unstructured correlation matrix will be used for the residuals. The estimated treatment effect at 120 day will be reported with a 95% confidence interval and corresponding p value. The main conclusion of the trial will be based on this analysis time point. We will also report the treatment effect at 30 days.

The analysis will include all available follow-up data. The model is likelihood based and missing data will be assumed to be missing-at-random (MAR), conditional on the observed values of other variables included in the analysis. If there are missing values for baseline measure of a clinical outcome, they will be replaced by the mean of the observed baseline values for all participants in all treatment arms (mean imputation). Sensitivity analyses will assess the validity of the MAR assumption and address the impact of missing data for all patients if required (see Section 9.8).

Model assumptions, including the normality of residuals and random effects distributions will be assessed. If any assumptions are poorly met, then an appropriate transformation of the outcome (e.g. log transformation) will be considered.

If the repeated measured mixed effects model including both 30 and 120 day outcomes fails to converge, treatment effects will be estimated using separate mixed linear regression models for each follow-up time point, which include participant as a random effect and fixed effects for treatment and period, type of AI (primary vs secondary), baseline osteocalcin and use of a bone sparing agent. An unstructured correlation matrix will be used for the residuals.

A carryover effect is not expected; therefore, the choice of a crossover design, the evaluation of efficacy by assessment of the change from trial baseline, and the proposed analysis model is considered to be appropriate. However, we will assess whether there is any evidence of a carry-over effect by fitting a separate linear mixed model which further includes the treatment by period interaction. In the case that there is a significant carry over effect we will fit a repeated measures linear mixed effect model using change from baseline in treatment period 1 (at 30 days and 120 days) as the dependent variable, a random participant effect, fixed effect for treatment, type of AI, baseline osteocalcin, use of bone sparing agent and time point. If this repeated measured model fails to converge, treatment effects for period 1 will be estimated using separate linear regression models for each follow-up time point, which include fixed effects for treatment, type of AI (primary vs secondary), baseline osteocalcin and use of a bone sparing agent.

9.6 SECONDARY EFFICACY ANALYSIS

9.6.1 OTHER MARKERS OF BONE HEALTH

Outcome definition: Treatment arm difference in i) P1NP, ii) BALP, iii) Corrected calcium, iv) PTH, v) Vitamin D, vi) NTX from baseline to Day 120.

Analysis: A multilevel repeated measures linear mixed effect model will be used to estimate the treatment arm difference for each of the above bone markers from baseline to Day 120. Similar to the primary analysis model, the model will include fixed effects for time (2 categories: Day 30 and Day 120), treatment arm (2 categories: prednisolone and hydrocortisone), period, type of AI (primary or secondary), baseline outcome, use of a bone sparing agent. A random participant effect will be included and an unstructured covariance matrix will be used. The treatment effect at 120 days will be reported with a 95% confidence interval. The models assumptions about random effects distributions, correlation structure and residuals will all be investigated. If any assumptions are poorly met then log transformation of the analyte in question, will be explored. If the mixed effect model including both 30 and 120 day outcomes fails to converge, treatment effects will be estimated using separate mixed linear regression models for each follow-up time point.

9.6.2 SURROGATE MARKERS AND RISK FACTORS FOR CARDIOVASCULAR DISEASE

Outcome definition: Treatment arm difference in i) Blood pressure, ii) Heart rate, iii) BMI, iv) Weight, v) Waist-hip circumference ratio, vi) NTX, vii) hs-CRP viii) Troponin I, ix) lipid profile, x)BNP; from baseline to Day 120.

Analysis: A multilevel repeated measures linear mixed effect model will be used to estimate the treatment arm difference in the above cardiovascular risk factors, from baseline to Day 120. Similar to the primary analysis model, the model will include fixed effects for time (2 categories: Day 30 and Day 120), treatment arm (2 categories: prednisolone and hydrocortisone), period, type of AI (primary or secondary), baseline outcome, use of a bone sparing agent. A random participant effect will be included and an unstructured covariance matrix will be used. The treatment effect at 120 days will be reported with a 95% confidence interval. The models assumptions about random effects distributions, correlation structure and residuals will all be investigated. If any assumptions are poorly met then log transformation of the analyte in question, will be explored. If the mixed effect model including both 30 and 120 day outcomes fails to converge, treatment effects will be estimated using separate mixed linear regression models for each follow-up time point.

9.6.3 GLYCAEMIC CONTROL

Outcome definition: Treatment arm difference in i) HbA1c, ii) Fructosamine, iii) fasting glucose levels, iv) insulin resistance (calculated using HOMA-IR); from baseline to Day 120.

Analysis: A multilevel repeated measures linear mixed effect model will be used to estimate the treatment arm difference in the above glycaemic control markers, from baseline to Day 120. Similar to the primary analysis model, the model will include fixed effects for time (2 categories: Day 30 and Day 120), treatment arm (2 categories: prednisolone and hydrocortisone), period, type of AI (primary or secondary), baseline outcome, use of a bone sparing agent. A random participant effect will be included and an unstructured covariance matrix will be used. The treatment effect at 120 days will be reported with a 95% confidence interval. The models assumptions about random effects distributions, correlation structure and residuals will all be investigated. If any assumptions are poorly met then log transformation of the analyte in question, will be explored. If the mixed effect model

including both 30 and 120 day outcomes fails to converge, treatment effects will be estimated using separate mixed linear regression models for each follow-up time point.

9.6.4 INFECTION RATES AND SEVERITY

Outcome definition: Treatment arm difference in the frequency of infections from baseline to Day 120.

Analysis: The GNCQ is a validated questionnaire that records the frequency of 6 different type of infections in the last 6 months (upper respiratory tract, lower respiratory tract, gastroenteritis, mucosal infections, lower urinary tract infections and flu). Respondents record either 0, 1, 2, 3, >3, or unknown frequencies of each type of infection.

The data will be tabulated and reported for each infection type at baseline and Day 120 for each treatment arm. Mcnemar-Bowkers test will be used to assess the change between treatment periods.

The data will also be weighted as follows: 0 or "don't know" will be scored as 0.5, 1-2 as 2, 3 or more as 4. The sum of the weighted scores will be treated as a continuous variable and a multilevel repeated measures linear mixed effect model will be used to estimate the treatment arm difference, from baseline to Day 120. Similar to the primary analysis model, the model will include fixed effects for time (2 categories: Day 30 and Day 120), treatment arm (2 categories: prednisolone and hydrocortisone), period, type of AI (primary or secondary), baseline weighted GNCQ, use of a bone sparing agent. A random participant effect will be included and an unstructured covariance matrix will be used. The treatment effect at 120 days will be reported with a 95% confidence interval. The models assumptions about random effects distributions, correlation structure and residuals will all be investigated. If any assumptions are poorly met then log transformation of the analyte in question, will be explored. If the mixed effect model including both 30 and 120 day outcomes fails to converge, treatment effects will be estimated using separate mixed linear regression models for each follow-up time point.

9.6.5 IMMUNOLOGY PROFILES

Outcome definition: Treatment arm difference in i) total white cells, ii) neutrophils, iii) lymphocytes, iv) eosinophils, v) basophils, vi) natural killer cells (including subtypes), vii) monocytes (including subtypes) viii) soluble immunological analytes; from baseline to Day 120.

Analysis: A multilevel repeated measures linear mixed effect model will be used to estimate the treatment arm difference in the above immunological markers, from baseline to Day 120. Similar to the primary analysis model, the model will include fixed effects for time (2 categories: Day 30 and Day 120), treatment arm (2 categories: prednisolone and hydrocortisone), period, type of AI (primary or secondary), baseline weighted GNCQ, use of a bone sparing agent. A random participant effect will be included and an unstructured covariance matrix will be used. The treatment effect at 120 days will be reported with a 95% confidence interval. The models assumptions about random effects distributions, correlation structure and residuals will all be investigated. If any assumptions are poorly met then log transformation of the analyte in question, will be explored. If the mixed effect model

including both 30 and 120 day outcomes fails to converge, treatment effects will be estimated using separate mixed linear regression models for each follow-up time point.

The soluble immunological markers and subtypes of natural killer cells and monocytes to be assessed have yet to be determined. They will very likely include but not be limited to $CD56^{+}/CD16^{+}$ NK cells, $CD56^{+}/CD16^{-}$ NK cells, $CD14^{+}/CD16^{-}$ monocytes, $CD14^{+}/CD16^{+}$ monocytes, $CD14^{-}/CD16^{+}$ monocytes and soluble CD16 antigen. The other specific markers to be assessed will be decided upon by the CI during the course of the study and will be based upon up to date published evidence.

9.6.6 SAFETY

Outcome definition: Treatment arm difference in i) FBC, ii) renal profile, iii) LFTs, iv) CK, v) ACTH, vi) CBG, vii) Bicarbonate viii) number of reports of myopathic symptoms, or nausea; from baseline to Day 120.

Analysis: A multilevel repeated measures linear mixed effect model will be used to estimate the treatment arm difference in the above safety outcomes (except (viii)), from baseline to Day 120. Similar to the primary analysis model, the model will include fixed effects for time (2 categories: Day 30 and Day 120), treatment arm (2 categories: prednisolone and hydrocortisone), period, type of AI (primary or secondary), baseline outcome. A random participant effect will be included and an unstructured covariance matrix will be used. The treatment effect at 120 days will be reported as a p-value with a 95% confidence interval. The models assumptions about random effects distributions, correlation structure and residuals will all be investigated. If any assumptions are poorly met then log transformation of the analyte in question, will be explored. If the mixed effect model including both 30 and 120 day outcomes fails to converge, treatment effects will be estimated using separate mixed linear regression models for each follow-up time point.

Data for symptom reporting will tabulated according frequency of reported symptoms will be presented at baseline and Day 120 for each treatment arm. McNemar's test will be used to assess the change between time periods.

9.6.7 WELLBEING

Outcome definition: Treatment arm difference in i) SF-36 score, ii) Addi-QoL score from baseline to Day 120.

Analysis: The SF-36 is scored according to the RAND Corporation scoring instructions. Briefly, the responses to questions are individually awarded a weighted score and are grouped according to the domain assessed. The scores for questions from a given domain are averaged, and a final score for the domain is produced (from 0 – 100, higher scores indicating a more positive outcome). The 8 domains assessed by the SF-36 include: physical functioning, role functioning/physical, role functioning/emotional, energy/fatigue, emotional wellbeing, social functioning, pain, general health and health change. Each domain will be analysed individually.

The Addi-QoL is a validated questionnaire containing 30 questions about wellbeing. Positive question responses are graded and weighted from 1 to 4, and negative question responses are graded from 4 to 1. The total score from the questionnaire will be between 30 and 120, with a higher value indicating a more positive outcome.

A multilevel repeated measures linear mixed effect model will be used to estimate the treatment arm difference in the above questionnaires, from baseline to Day 120. Similar to the primary analysis model, the model will include fixed effects for time (2 categories: Day 30 and Day 120), treatment arm (2 categories: prednisolone first and hydrocortisone first), period, type of AI, baseline measurement and use of a bone sparing agent. A random participant effect will be included and an unstructured covariance matrix will be used. The treatment effect at 120 days will be reported as a p-value with a 95% confidence interval. The models assumptions about random effects distributions, correlation structure and residuals will all be investigated. If any assumptions are poorly met then log transformation of the analyte in question, will be explored. If the mixed effect model including both 30 and 120 day outcomes fails to converge, treatment effects will be estimated using separate mixed linear regression models for each follow-up time point.

9.6.8 COMPLIANCE TO REGIMEN

Outcome definition: Treatment arm difference in the number of tablets returned between Day 120 of each treatment period.

Analysis: The number of tablets returned by each patient at the end of each treatment period will be counted and recorded. The number of tablets expected back will be calculated from the patients dosing diary, number of tablets dispensed, and any known losses of tablets reported by the patient. The difference between the expected number of tablet and actual number of tablets received will correspond to the number of missed doses.

The number of missed doses in each treatment period will be tested for normality using the Shapiro-Wilk test. If the assumption of normality is not met, then a log transformation will be explored. A paired t-test will be used to assess the mean difference in missed doses between treatment periods. Where the data is non-parametric, a Wilcoxon test will be used.

9.7 SAFETY ANALYSIS

A safety set (SS) population, consisting of all participants who received at least one dose of the assigned intervention, will be defined for describing adverse events in. Adverse events will be summarised by type (AE or SAE) and treatment arm in a given period. AEs will be tabulated by treatment group for both the number of events and the number of participants with the type of event in a given period. A listing will be produced detailing all Serious Adverse Events (SAEs). Only descriptive analysis of safety will be performed, no formal statistical testing is planned.

9.8 SENSITIVITY ANALYSIS

Every effort will be made to obtain follow up date for participants including those that stop treatment. The primary analysis method outlined above employs maximum likelihood estimation and thus is efficient for handing missing outcome data under a Missing-at-Random (MAR) assumption. That is, it assumes the probability of missing data is not dependent on the values of the unobserved data themselves, conditional on the observed values of the variables included in the analysis model. If >10% individuals are missing the primary outcome at 120 days, Sensitivity analyses will explore the impact of departures from the main MAR assumption on the primary outcome using a pattern-mixture modelling and multiple imputation approach (11). Imputation under MAR will initially be performed separately within each treatment arm using chained equations following the guidance suggested by White et al (12). The variables in the imputation model will be the same as those in the analysis model without including more auxiliary variables after taking into account of the relative small sample size of this study. Imputations will then be modified to reflect departures from the MAR assumption.

We will investigate the impact of a better or poorer response than that predicted by MAR (lower/higher bone turnover) for participants with missing data. To do so we will define δ as the postulated mean difference in the change of bone turnover between the observed and unobserved cases. For each participant in each intervention arm we then modify the MAR imputed observations accordingly by δ. Imputed data sets will be analysed using the primary analysis model. Results will be combined across imputed data sets using Rubin's rules. We will repeat the analysis for a range of δ corresponding to +/- 10, 20, 30, 40 and 50% of the change of bone turn over 120 days in all observed participants.

9.9 SUPPLEMENTARY ANALYSIS

9.9.1 Pre-protocol analysis

The primary analysis will be repeated on the per-protocol sub-group to evaluate the effect of deviations on the primary results.

9.10 SUBGROUP ANALYSIS

Three subgroup analyses are planned for the primary endpoint, and markers of cardiovascular disease, to investigate whether the intervention effect differ in patients:

1) Taking bone sparing agents
2) Taking anti-hypertensives
3) Taking lipid-lowering agents

All subgroup analyses will be undertaken using the primary analysis model. For each subgroup factor the model will be updated to include an additional covariate for the subgroup factor (where required) and the subgroup by treatment group interaction term.

10.0 REFERENCES

(1) Erichsen MM, Lovas K, Fougner KJ, Svartberg J, Hauge ER, Bollerslev J, et al. Normal overall mortality rate in Addison's disease, but young patients are at risk of premature death. European journal of endocrinology / European Federation of Endocrine Societies. 2009;160(2): 233-237.

(2) Bornstein SR, Allolio B, Arlt W, Barthel A, Don-Wauchope A, Hammer GD, et al. Diagnosis and Treatment of Primary Adrenal Insufficiency: An Endocrine Society Clinical Practice Guideline. The Journal of clinical endocrinology and metabolism. 2016;101(2): 364-389.

(3) Iqbal K, Halsby K, Murray RD, Carroll PV, Petermann R. Glucocorticoid management of adrenal insufficiency in the United Kingdom: assessment using real-world data. Endocrine connections. 2019;8(1): 20-31.

(4) Shenfield GM, Paterson JW, Costello JF, Ijaduola O. The effect of prednisone treatment on the half-life of intravenous hydrocortisone. British journal of clinical pharmacology. 1974;1(3): 237-240.

(5) Koetz KR, Ventz M, Diederich S, Quinkler M. Bone mineral density is not significantly reduced in adult patients on low-dose glucocorticoid replacement therapy. The Journal of clinical endocrinology and metabolism. 2012;97(1): 85-92.

(6) Hunt PJ, Gurnell EM, Huppert FA, Richards C, Prevost AT, Wass JA, et al. Improvement in mood and fatigue after dehydroepiandrosterone replacement in Addison's disease in a randomized, double blind trial. The Journal of clinical endocrinology and metabolism. 2000;85(12): 4650-4656.

(7) Benson S, Neumann P, Unger N, Schedlowski M, Mann K, Elsenbruch S, et al. Effects of standard glucocorticoid replacement therapies on subjective well-being: a randomized, double-blind, crossover study in patients with secondary adrenal insufficiency. European journal of endocrinology. 2012;167(5): 679-685.

(8) Oksnes M, Bensing S, Hulting AL, Kampe O, Hackemann A, Meyer G, et al. Quality of life in European patients with Addison's disease: validity of the disease-specific questionnaire AddiQoL. The Journal of clinical endocrinology and metabolism. 2012;97(2): 568-576.

(9) Committee for Medicinal Products for Human Use (CHMP). Guideline on adjustment for baseline covariates in clinical trials. EMA/CHMP/295050/2013 ed. European Medicines Agency; 2015.

(10) White IR, Thompson SG. Adjusting for partially missing baseline measurements in randomized trials. Statistics in medicine. 2005;24(7): 993-1007.

(11) Carpenter J, Kenward M. Missing data in randomised controlled trials—a practical guide. . London School of Hygiene: 2007.

(12) White IR, Royston P, Wood AM. Multiple imputation using chained equations: Issues and guidance for practice. Statistics in medicine. 2011;30(4): 377-399.

11.0 APPENDICES

11.1 APPENDIX 1

Appendix 1: schematic of study periods and visits Participants will attend visit 1 on their baseline therapy in both periods. They will be issued with steroid/placebo tablets that are identical in appearance. On Day 2, they will be instructed to take the tablets at the stated set times for a period of 4 months in the first period. This is followed by a 2-week minimum washout period on their baseline steroid replacement therapy, before commencing the second period. Patients will be on baseline therapy for Visit 1 of the second period prior to starting the blinded therapy on Day 2 for 4 months. Patients will randomised to undertake either arm (A) or (B). Arm (A) will involve administration of prednisolone in the first period and hydrocortisone in the second. Arm (B) will involve hydrocortisone in the first period and prednisolone in the second period. The example schematic below is for a participant on arm (A).

Study Period	Drug- Arm (A)	On Waking	Lunchtime (1200h)	Late afternoon (1700h)
1	Prednisolone	2-5mg	Placebo	Placebo
2	Hydrocortisone	10mg	5mg	5mg

First Study Period (e.g. Arm (A)- Prednisolone/Placebo/Placebo)

Time	Study Visit 1<br>Month 1<br>Day 1	Month 1<br>Day 2	Telephone Consult 1<br>Month 1<br>Day 8	Telephone Consult 2<br>Month 1<br>Day 15	Study Visit 2<br>Month 1<br>Day 30	Telephone Consult 3<br>Month 2<br>Day 60	Telephone Consult 4<br>Month 3<br>Day 90	Study Visit 3<br>Month 4<br>Day 120	
06:00	Patient takes 1st tablet of the day at home (Baseline therapy)	Patient starts blinded medication at home			Patient takes 1st tablet of the day at home (Blinded treatment)			Patient takes 1st tablet of the day at home (Blinded treatment)	2-week washout period on baseline therapy
08:00	Record weight, BP, heart rate, waist and hip circumference				Record weight, BP, heart rate, waist and hip circumference			Record weight, BP, heart rate, waist and hip circumference	
08:20	Obtain urine sample (NTX & pregnancy test)				Obtain urine sample (NTX & pregnancy test)			Obtain urine sample (NTX & pregnancy test)	
08:40	Blood profile 1				Blood profile 1			Blood profile 1	
08:55	AE reporting		AE reporting	AE reporting	AE reporting	AE reporting	AE reporting	AE reporting	
09:15	SHS surveys/GNCQ				SHS surveys/GNCQ			SHS surveys/GNCQ	

Second Study Period (e.g. Arm (A)- Hydrocortisone 10mg / 5mg / 5mg)									
**Time**	**Study Visit 1 (4)** **Month 1** **Day 1**	**Month 1** **Day 2**	**Telephone Consult 1 (5)** **Month 1** **Day 8**	**Telephone Consult 2 (6)** **Month 1** **Day 15**	**Study Visit 2 (5)** **Month 1** **Day 30**	**Telephone Consult 3 (7)** **Month 2** **Day 60**	**Telephone Consult 4 (8)** **Month 3** **Day 90**	**Study Visit 3 (6)** **Month 4** **Day 120**	**Study complete (Patient returns to baseline therapy)**
06:00	Patient takes 1st tablet of the day at home (Baseline therapy)	Patient starts blinded medication at home			Patient takes 1st tablet of the day at home (Blinded treatment)			Patient takes 1st tablet of the day at home (Blinded treatment)	
08:00	Record weight, BP, heart rate, waist and hip circumference				Record weight, BP, heart rate, waist and hip circumference			Record weight, BP, heart rate, waist and hip circumference	
08:20	Obtain urine sample (NTX & pregnancy test)				Obtain urine sample (NTX & pregnancy test)			Obtain urine sample (NTX & pregnancy test)	
08:40	Blood profile 1				Blood profile 1			Blood profile 1	
08:55	AE reporting		AE reporting	AE reporting	AE reporting	AE reporting	AE reporting	AE reporting	
09:15	SHS surveys/GNCQ				SHS surveys/GNCQ			SHS surveys/GNCQ	

AE: Adverse Event; **SHS**:Subjective Health Status (including the short form health survey (SF-36) and AddiQoL); **GNCQ**: German National Cohort Questionnaire; **Blood profile 1**:fasting renal, bone and lipid profiles, bicarbonate, full blood count (FBC), glucose, insulin, fructosamine, HbA1c, creatine kinase(**CK**), Adrenocorticotropic Hormone (ACTH), cortisol binding globulin (CBG), parathyroid hormone (PTH), vitamin D, bone-specific alkaline phosphatase (**BALP**), osteocalcin (OC), procollagentype1 N-terminal propeptide(**P1NP**); hs-CRP, hs-Troponin I, BNP, assessment of soluble immunological antigens and assessment of white cell populations.
