# Supplementary material for: Prednisolone Once Daily vs Hydrocortisone Thrice Daily in Hypoadrenalism: A Randomized Clinical Trial
Source: JAMA Netw Open. 2026 Mar 24;9(3):e262982. doi: 10.1001/jamanetworkopen.2026.2982 (PMC13014172; doi:10.1001/jamanetworkopen.2026.2982)
Supplement: Supplement 2. — eMethods. Supplementary methods eTable 1. Assay platform and performance specification for analytes measured at North West London Pathology (NWLP) eTable 2. Aetiology of adrenal insufficiency in study participants eTable 3. Frequency of steroid regimens used during the main study eTable 4. Extended outcome data of all secondary outcomes eTable 5. Tabulation of all adverse events according to concurrent glucocorticoid at the time [file jamanetwopen-e262982-s002.pdf]

Supplemental Online Content

Choudhury S, Lazarus K, Sharma A, et al. Prednisolone once daily vs hydrocortisone thrice daily in hypoadrenalism: a randomized clinical trial. *JAMA Netw Open*. 2026;9(3):e262982. doi:10.1001/jamanetworkopen.2026.2982

eMethods. Supplementary methods

eTable 1. Assay platform and performance specification for analytes measured at North West London Pathology (NWLP)

eResults. Supplementary results

eTable 2. Aetiology of adrenal insufficiency in study participants

eTable 3. Frequency of steroid regimens used during the main study

eTable 4. Extended outcome data of all secondary outcomes

eTable 5. Tabulation of all adverse events according to concurrent glucocorticoid at the time

This supplemental material has been provided by the authors to give readers additional information about their work.

© 2026 Choudhury S et al. *JAMA Network Open*.

eMethods. Supplementary methods

Trial summary:

This trial is a phase 3, two-arm, two-period blind cross-over study. Participants were randomised to one of two arms:

a) Prednisolone first, in which individuals were given low-dose prednisolone (2 mg – 5mg) in the first study period followed by hydrocortisone in the second study period.
b) Hydrocortisone first, in which individuals received standard regimens of hydrocortisone in the first study period and low-dose prednisolone in the second study period.

The two study periods were 120 days in duration each, and were interrupted by a minimum 2-week washout period in which participants returned to open-label baseline glucocorticoid replacement medication. Baseline data for each study period was collected on Day 1. Outcome data was collected on Day 30 and Day 120. All data was collected in stereotyped study visits, which were conducted at the same time for each participants, in the morning.

The specific dosing and timing of the blinded study medication was the same as the patient's baseline pre-study treatment. The handling of participants who had not experienced both hydrocortisone and prednisolone treatment prior to the study has been discussed in the primary paper and elaborated on below. Blinding was maintained by providing single hydrocortisone tablets containing the correct dose as per the participant's individualised regimen during the hydrocortisone period, and providing a single low-dose prednisolone tablet in the morning, followed by single placebo tablets at noon and in the afternoon when the participant was on the prednisolone period. All medication was taken as single tablets containing tailored doses.

Glucocorticoid dose optimisation:

Where participants were naïve to either one of prednisolone or hydrocortisone treatment, they were trialled on the medication in question and titrated up or down as per routine clinical care at Imperial College Healthcare NHS Trust. This process was completed prior to randomisation, to ascertain the dose to be taken forwards into the study.

If a participant was naïve to prednisolone, they would usually be commenced on 4 mg of prednisolone once daily. At the patient's convenience, usually after a few days, an 8-hour serum prednisolone level would be measured. The target range is 15 – 25 µg/L. Where participants were above this range, they would be asked to reduce the dose by 1 mg. If they were below the range, they would be asked to increase the dose by 1 mg. After each change, a repeat 8-hour level would be measured to ensure they were in the target range. Once a patient had settled on a dose for a minimum 2-week period and were clinically well, this dose was taken forwards into the main study,

In the case of hydrocortisone, the patient would be commenced on 10 mg + 5 mg + 2.5 mg. A hydrocortisone day curve was completed if needed and the dose titrated up or down accordingly. Cortisol levels were measured pre-dose and 1 hour post dose. Target ranges were as follows: 350 – 600 nmol/L with the morning dose, 200 – 300 nmol/L with the second dose and 100 – 200 nmol/L with the third dose. The hydrocortisone regimen was modified according to the results. The participants were required to be clinically well on the final hydrocortisone regimen for a minimum of 2 weeks, prior to this dose being taken forwards into the main study,

Outcome measures:

The primary outcome measure was assessment of bone turnover by quantifying levels of carboxylated and undercarboxylated osteocalcin (Gla-OC and Glu-OC, respectively). Secondary outcomes included:

- Other markers of bone health assessed by measurement of change in additional bone markers and bone profile including procollagen type-1 N-terminal propeptide (P1NP), bone specific alkaline phosphatase

© 2026 Choudhury S et al. *JAMA Network Open*.

(BALP), corrected calcium, parathyroid hormone (PTH), vitamin D and urinary N-terminal telopeptide (NTX).

- Surrogate markers and risk factors for cardiovascular disease including anthropometric markers such as: blood pressure, heart rate, BMI (height on first occasion), weight and waist-hip circumference ratio.
- Cardiovascular risk assessed by measurement of high-sensitivity CRP, high-sensitivity troponin I and BNP.
- Glycaemic control assessed by HbA1c, fructosamine, fasting glucose levels and insulin resistance represented by HOMA-IR
- Infection rates and severity assessed by completion of the German National Cohort Questionnaire (GNCQ)
- Immunology profiles assessed by measurement and assessment of soluble immunological analytes and isolated white cell populations.
- Safety assessed by reporting of symptoms of steroid deficiency and myopathy and review of routine monitoring blood tests including full blood count (FBC), renal profile, liver function tests (LFTs), creatine kinase (CK), Adrenocorticotropic hormone (ACTH) cortisol binding globulin (CBG) and bicarbonate.
- Wellbeing assessed by subjective health questionnaires including the SF-36 and Addi-QoL
- Compliance to regimen assessed by collecting the remaining unused tablets at the end of each treatment arm

Inclusion/Exclusion Criteria

Inclusion and exclusion criteria have been outlined in the study protocol.

Assays

Osteocalcin, both Gla-OC and Glu-OC were both measured using commercial enzyme linked immunosorbent assays (ELISAs) (Takara Bio, Saint-Germain-en-Laye, France). The Gla-OC assay demonstrated a lower limit of quantification (LLoQ) of 0.5 ng/ml, with a reported inter- and intra- assay coefficient of variation (CV) of <2.4% and <4.8% respectively. The Glu-OC assay has a LLOQ of 0.25 ng/ml with inter- and intra- assay imprecision of <6.7% and <9.9% respectively.

All other analytes were measured by pathology services at North West London Pathology (NWLP). All specimens were collected in keeping with sample collection requirements published by NWLP. The majority of analyte quantification was performed on the Abbott Alinity platform. The assay platforms used and performance specification are outlined in eTable 1.

eTable 1. Assay platform and performance specification for analytes measured at North West London Pathology (NWLP)

Assay	Platform	Lower Limit of Quantification (LLOQ)	Inter-assay CV	Intra-assay CV
P1NP	Roche Cobas	5 ng/ml	<3.7%	<3.2%
Urine NTX	Commercial Osteomark ELISA	1 BCE nmol/L	<5.0%	<19.0%
Creatinine (urine)	Abbott Alinity	0.442 mmol/L	<2.0%	<1.7%
Calcium	Abbott Alinity	0.25 mmol/L	<1.0%	<0.6%
Albumin	Abbott Alinity	3.1 g/L	<1.2%	<0.4%
Parathyroid hormone	Abbott Alinity	0.3 pmol/L	<8.7% (Total CV)	<8.7%
25-OH Vitamin D	Abbott Alinity	8.5 mmol/L	<7.1%	<5.1%
Creatinine (serum)	Abbott Alinity	17.7 µmol/L	<1.9%	<1.5%
Potassium	Abbott Alinity	1.0 mmol/L	<1.2%	<0.7%

© 2026 Choudhury S et al. *JAMA Network Open*.

Bicarbonate	Abbott Alinity	4 mmol/L	<3.9%	<2.2%
High Sensitivity Troponin	Abbott Alinity	2 ng/L	<7.2%	<5.0%
High Sensitivity CRP	Abbott Alinity	0.1 mg/L	<4.00%	<2.38%
BNP	Abbott Alinity	10 ng/L	<6.7% (Total CV)	<5.6%
HbA1c	Tosoh G11	3 mmol/mol (<20mmol/mol not routinely reported)	<2.6% (Total CV)	<2.4%
Fructosamine	Sent to City Assays, Black Country Pathology Services, UK: In house colorimetric assay	10 mmol/L	<2.5%	<1.0%
HDL	Abbott Alinity	0.13 mmol/L	<5.1%	<1.7%
Triglycerides	Abbott Alinity	0.071 mmol/L	<1.7%	<0.8%
Cholesterol	Abbott Alinity	0.18 mmol/L	<1.4%	<1.1%
Glucose	Abbott Alinity	0.278 mmol/L	<0.99%	<1.98%
Insulin	Abbott Alinity	1.0 mIU/L	<5.2%	<4.2%
C-peptide	Abbott Alinity	3.31 pmol/L	<4.0% (Total CV)	<2.4%
FBC	Sysmex XE2100	N/A	N/A	N/A
ACTH	Siemens Immulite	5 ng/L	<10.0%	<9.5%
Cortisol	Abbott Alinity	28 nmol/L	<6.2%	<5.5%
Prednisolone	In-house HPLC-MS/MS	10 µg/L	<6.5%	<6.5%

Fructosamine samples were handled by NWLP, but were sent away to another provider for quantification. All other analysis was completed in house by NWLP, as specified. Abbreviations: Coefficient of Variation (CV); Procollagen Type-1 N-Terminal Propeptide (P1NP); N-Terminal Telopeptide (NTX); Brain Natriuretic Peptide (BNP); High Density Lipoproteins (HDL); Full Blood Count (FBC); Adrenocorticotropic Hormone (ACTH).

eResults. Supplementary results

The aetiology of adrenal insufficiency seen in the study population has been tabulated in eTable 2.

eTable 2. Aetiology of adrenal insufficiency in study participants

Main Diagnosis	Frequency	Relevant Secondary Diagnosis	Frequency
**Primary Adrenal Insufficiency:**			
Addison's Disease	10		
Bilateral Adrenalectomy	5		
		Cushing's Disease	2
		CAH	1
		Ectopic Cushing's Syndrome	1
		Von Hippel Lindau	1
Unilateral Adrenalectomy	1		
		Adrenal Cushing's Syndrome	1
**Total**	**16**		
**Secondary Adrenal Insufficiency:**			

© 2026 Choudhury S et al. *JAMA Network Open*.

Non Functioning Adenoma	7		
Cushing's Disease	5		
Acromegaly	4		
Craniopharingioma	3		
Macroadenoma	3		
Rathke's Cyst	2		
Pituitary Apoplexy	2		
Macroprolactinoma	1		
Sheehan's Syndrome	1		
Cranial radiotherapy for facial sarcoma	1		
Brain tumour secondary to B-cell Lymphoma	1		
FSH-oma	1		
**Total**	**31**		
Abbreviations: Follicle stimulating Hormone (FSH)			

The frequency of glucocorticoid regimens used in the study have been recorded in eTable 3.

eTable 3. Frequency of steroid regimens used during the main study

**Steroid Regimen:**	**Frequency**
Hydrocortisone:	
10 mg +5 mg + 5 mg	22
10 mg + 5 mg + 2.5 mg	10
10 mg + 5 mg	4
10 mg +10 mg	4
20 mg +10 mg + 5mg	1
20 mg + 5mg + 5 mg	1
15 mg + 5 mg + 2.5 mg	2
15 mg + 10 mg + 2.5 mg	1
10 mg + 5 mg + 5 mg + 2.5 mg	1
**Total**	46
Prednisolone:	
2 mg	4
3 mg	19
4 mg	22
5 mg	1
**Total**	46
Median (IQR) hydrocortisone dose used: 20 mg (17.5 – 20 mg). Median (IQR) dose of prednisolone used: 3.5 mg (3 – 4 mg)	

Secondary outcome data results have been tabulated in eTable 4. All adverse events have been summarised in eTable 5.

eTable 4. Extended outcome data of all secondary outcomes

Parameter	Day 30 outcomes				Day 120 Outcomes			
	**HC treatment effect from baseline**	**Pred treatment effect from baseline**	**Difference of treatment effect (Pred – HC)**	**P-value**	**HC treatment effect from baseline**	**Pred treatment effect from baseline**	**Difference of treatment effect (Pred – HC)**	**P-value**
Fractional excretion of Sodium	84.0 (7.5 to 160.4)	5.2 (-71.3 to 81.7)	-78.8 (-178 to 20.3)	0.12	134.6 (58.4 to 211.0)	25.1 (-51.1 to 101.0)	-109 (-195 to -24.2)	*0.01
Free water excretion (1- Fe_{Osm})	0.09 (0.00 to 0.18)	-0.05 (-0.14 to 0.04)	-0.14 (-0.26 to -0.02)	*0.02	0.08 (-0.01 to 0.17)	-0.06 (-0.15 to 0.03)	-0.139 (-0.25 to -0.02)	*0.02
High Sensitivity CRP (mg/L)	-0.86 (-1.49 to -0.24)	-0.31 (-0.94 to 0.31)	0.55 (-0.16 to 1.26)	0.13	-0.62 (-1.73 to 0.49)	-0.41 (-1.52 to 0.70)	0.21 (-0.24 to 0.66)	0.36
Brain Natriuretic Peptide (ng/L)	2.67 (-2.93 to 8.28)	-1.17 (-6.79 to 4.45)	-3.84 (-9.21 to 1.52)	0.16	0.81 (-5.01 to 6.71)	-1.86 (-7.77 to 4.05)	-2.67 (-9.18 to 3.84)	0.41
Total Cholesterol (mmol/L)	0.01 (-0.15 to 0.17)	-0.03 (-0.19 to 0.13)	-0.04 (-0.26 to 0.19)	0.75	0.14 (-0.02 to 0.29)	0.05 (-0.11 to 0.20)	-0.09 (-0.30 to 0.12)	0.40
Triglycerides (mmol/L)	0.08 (-0.10 to 0.25)	-0.06 (-0.24 to 0.12)	-0.13 (-0.67 to 0.10)	0.26	-0.02 (0.15 to 0.11)	-0.05 (-0.17 to 0.08)	-0.02 (-0.20 to 0.15)	0.80
HDL (mmol/L)	0.00 (-0.05 to 0.05)	-0.01 (-0.06 to 0.04)	-0.02 (-0.08 to 0.04)	0.57	0.05 (-0.01 to 0.11)	-0.01 (-0.06 to 0.05)	-0.06 (-0.14 to 0.02)	0.14
LDL (mmol/L)	0.00 (-0.13 to 0.13)	0.02 (-0.10 to 0.15)	0.02 (-0.15 to 0.20)	0.79	0.09 (-0.03 to 0.22)	0.08 (-0.05 to 0.20)	-0.02 (-0.19 to 0.16)	0.83
Non-HDL (mmol/L)	0.00 (-0.14 to 0.15)	-0.03 (-0.17 to 0.12)	-0.03 (-0.23 to 0.17)	0.76	0.08 -0.05 to 0.22)	0.05 (-0.09 to 0.18)	-0.04 (-0.22 to 0.16)	0.71
White blood cells (x10^9 /L)	-0.44 (-0.80 to -0.08)	-0.29 (-0.65 to 0.07)	0.15 (-0.32 to 0.63)	0.52	0.01 (-0.40 to 0.43)	-0.08 (-0.50 to 0.34)	-0.09 (-0.62 to 0.44)	0.73
Neutrophils (x10^9 /L)	-0.43 (-0.77 to -0.09)	-0.14 (-0.48 to 0.19)	0.29 (-0.14 to 0.71)	0.18	-0.10 (-0.52 to 0.32)	0.00 (-0.42 to 0.42)	0.09 (-0.43 to 0.61)	0.72
Lymphocytes (x10^9 /L)	0.02 (-0.07 to 0.11)	-0.06 (-0.14 to 0.03)	-0.07 (-0.20 to 0.05)	0.22	0.05 (-0.07 to 0.17)	-0.02 (-0.14 to 0.10)	-0.06 (-0.23 to 0.10)	0.43
Eosinophils (x10^9 /L)	-0.02 (-0.05 to 0.01)	-0.02 (-0.05 to 0.00)	-0.01 (-0.05 to 0.03)	0.68	-0.01 (-0.04 to 0.02)	0.00 (-0.03 to 0.03)	0.00 (-0.03 to 0.04)	0.80
Basophils (x10^9 /L)	-0.01 (0.02 to 0.00)	-0.01 (-0.02 to 0.00)	0.00 (-0.01 to 0.01)	0.97	-0.01 (-0.02 to 0.00)	-0.01 (-0.02 to 0.00)	0.00 (-0.02 to 0.02)	0.97
Monocytes (x10^9 /L)	0.02 (-0.01 to 0.01)	-0.02 (-0.05 to 0.01)	-0.04 (-0.08 to -0.01)	*0.02	0.04 (0.00 to 0.01)	0.01 (-0.03 to 0.05)	-0.03 (-0.08 to 0.02)	0.28
Alanine aminotransferase	2.80 (-0.85 to 6.44)	2.37 (-1.28 to 6.01)	-0.43 (-5.46 to 4.6)	0.86	0.58 (-1.93 to 3.09)	0.65 (-1.86 to 3.16)	0.07 (-2.58 to 2.72)	0.96

© 2026 Choudhury S et al. *JAMA Network Open*.

(U/L)								
Alkaline Phosphatase ((U/L)	0.40 (-2.61 to 3.41)	-0.51 (-3.52 to 2.51)	-0.91 (-5.06 to 3.25)	0.66	0.44 (-2.85 to 3.46)	0.80 (-2.23 to 3.83)	0.36 (-3.73 to 4.44)	0.86
Bilirubin (µmol/L)	-0.03 (-1.10 to 1.03)	0.53 (-0.54 to 1.60)	0.57 (-0.81 to 2.04)	0.44	0.11 (-1.04 to 1.25)	0.37 (-0.77 to 1.51)	0.27 (-1.2 to 1.74)	0.72
Creatine Kinase (U/L)	-27.9 (-46.0 to 9.7)	-17.4 (-35.5 to 0.7)	10.5 (-14.3 to 35.3)	0.40	-14.1 (-58.9 to 30.6)	1.4 (-43.3 to 46.0)	15.5 (-42.3 to 73.4)	0.59
ACTH (ng/L)	-79.9 (-143.0 to -15.5)	-48.1 (-112.1 to 15.6)	31.2 (-56.7 to 119)	0.48	-95.4 (-255.0 to 64.3)	41.1 (-118.0 to 200.3)	-137.0 (-83.2 to 356)	0.22
CBG (mg/L)	-1.35 (-3.91 to 1.21)	-0.59 (-3.16 to 1.99)	0.76 (-2.1 to 3.62)	0.59	-0.63 (-3.42 to 2.17)	-1.46 (-4.27 to 1.35)	-0.83 (-4.71 to 3.04)	0.67
Bicarbonate (mmol/L)	-0.23 (-0.91 to 0.45)	-0.99 (-1.67 to -0.31)	-0.76 (-1.65 to 0.14)	0.09	-0.93 (-1.65 to -0.21)	-0.48 (-1.20 to 0.25)	-0.45 (-0.54 to 1.45)	0.36
GNCQ:								
URTI	-0.16 (-0.43 to 0.12)	0.02 (-0.25 to 0.29)	0.18 (-0.09 to 0.44)	0.19	0.05 (-0.30 to 0.39)	-0.06 (-0.41 to 0.29)	-0.11 (-0.52 to 0.31)	0.61
GI	-0.14 (-0.27 to 0.00)	-0.07 (-0.20 to 0.06)	0.06 (-0.05 to 0.18)	0.26	-0.04 (-0.20 to 0.12)	-0.02 (-0.18 to 0.14)	0.02 (-0.20 to 0.23)	0.88
Skin	-0.20 (-0.38 to -0.01)	-0.33 (-0.53 to 0.14)	-0.14 (-0.31 to 0.04)	0.13	-0.59 (-0.77 to -0.41)	-0.59 (-0.77 to -0.40)	0.00 (-0.14 to 0.14)	0.96
Boils	-0.34 (-0.50 to -0.19)	-0.49 (-0.63 to 0.35)	-0.14 (-0.30 to 0.01)	0.06	-0.55 (-0.69 to -0.41)	-0.64 (-0.77 to -0.51)	-0.09 (-0.18 to 0.01)	0.07
UTI	-0.17 (-0.28 to -0.07)	-0.17 (-0.28 to 0.07)	0.00 (-0.08 to 0.08)	0.97	-0.43 (-0.61 to -0.26)	-0.50 (-0.68 to -0.32)	-0.07 (-0.20 to 0.07)	0.30
Flu	-0.16 (-0.36 to 0.04)	-0.11 (-0.32 to 0.09)	0.05 (-0.22 to 0.31)	0.71	0.05 (-0.18 to 0.28)	-0.07 (-0.30 to 0.16)	0.11 (-0.43 to 0.21)	0.48
Treatment effects estimated using repeated measures linear mixed model at Day 30 and Day 120 for hydrocortisone and prednisolone, with treatment differences. Treatment effects are represented as estimated marginal mean (95% confidence interval). *- P-values of <0.05. Abbreviations: German National Cohort Questionnaire (GNCQ), Upper Respiratory Tract Infection (URTI), Gastrointestinal (GI), Urinary Tract Infection (UTI).								

eTable 5. Tabulation of all adverse events according to concurrent glucocorticoid at the time

Adverse Event Term	Total number of events	During Study Period		During pre-study period or washout period	
		Number of events On Prednisolone	Number of events on Hydrocortisone	Number of events On Prednisolone	Number of evets on Hydrocortisone
Viral Illness	34	18	16		
Lethargy	12	7	5		
COVID-19	8	3	4	1	
Vaccination Complication	7	5	2		

© 2026 Choudhury S et al. *JAMA Network Open*.

Nausea	7	5	2		
Migraine	6	5	1		
Diarrhoea	6	2	4		
Viral Gastroenteritis	3 + 1 (SAE)	2 + 1 (SAE)	1		
URTI	3		3		
Iron Deficiency Anaemia	3	1	1		1
LRTI	2	1	1		
UTI	2		1	1	
Fatigue	2	1	1		
Gastric Reflux	2	2			
Tooth Infection	2		2		
Headache	2	1	1		
Acute Gastroenteritis	1 (SAE)	1 (SAE)			
Hyponatraemia	1 (SAE)				
Posterior Vitreous Detachment	1	1			
Eustachian Tube Dysfunction	1	1			
Parotitis	1	1			
Pre-syncope	1	1			
Asthma Exacerbation prophylaxis	1	1			
TMJ dysfunction	1			1	
Insect Bite	1	1			
General Body Pain	1		1		
Tinnitus	1		1		
Back Pain	1			1	
Constipation	1		1		
Muscle Strain	1		1		
Panic Attack	1	1			
Dyspepsia	1		1		
Nocturia	1		1		
Tooth Extraction	1		1		
Dental Implant	1		1		
Diverticulosis	1		1		
Surgical Complication	1				1
Food Poisoning	1		1		
Foot Pain	1		1		
Sore Throat	1		1		
Road Traffic Accident	1	1			
Biliary Colic	1	1			
Pneumonia	1	1			

© 2026 Choudhury S et al. *JAMA Network Open*.

Tonsilitis	1	1			
Light-headedness	1		1		
Myoglobinuria	1		1		
Facial Injury	1	1			
**Total AEs**	**133 (including 3 SAEs)**	**67 (including 3 SAEs)**	**60**	**4**	**2**
Doubled Steroids outside AE	17	5	10	1	1
**Total Events**	**150**	**72**	**70**	**5**	**3**
The total number of AEs and adverse events are balanced between the prednisolone and hydrocortisone. All SAEs were seen while participants were receiving prednisolone. Abbreviations: Coronavirus Disease 2019 (COVID-19), Upper Respiratory Tract Infection (URTI), Lower Respiratory Tract Infection (LRTI), Urinary Tract Infection (UTI), Adverse Event (AE), Serious Adverse Event (SAE)					

© 2026 Choudhury S et al. *JAMA Network Open*.
