# Supplementary material for: Prednisolone Once Daily vs Hydrocortisone Thrice Daily in Hypoadrenalism: A Randomized Clinical Trial
Source: JAMA Netw Open. 2026 Mar 24;9(3):e262982. doi: 10.1001/jamanetworkopen.2026.2982 (PMC13014172; doi:10.1001/jamanetworkopen.2026.2982)
Supplement: Supplement 3. — Data Sharing Statement [file jamanetwopen-e262982-s003.pdf]

Data Sharing Statement

Choudhury. Prednisolone Once Daily vs Hydrocortisone Thrice Daily in Hypoadrenalism. *JAMA Netw Open*. Published March 24, 2026. doi:10.1001/jamanetworkopen.2026.2982

Data
Additional Information: ClinicalTrials.gov https://clinicaltrials.gov/study/NCT03936517 NCT03936517
Data available: Yes
Data types: Other (please specify)
Additional Information: Some or all datasets generated during and/or analysed during the current study are not publicly available but are available from the corresponding author on reasonable request.
How to access data: Individual requests are to be sent to s.choudhury@imperial.ac.uk
When available: With publication

Supporting Documents
Document types: None

Additional Information
Who can access the data: To individuals who have emailed reasonable requests
Types of analyses: This will depend on the request.
Mechanisms of data availability: With investigator support
